# Viral Metagenomic Content Reflects Seawater Ecological Quality in the Coastal Zone

**DOI:** 10.3390/v12080806

**Published:** 2020-07-26

**Authors:** Anastasia Tsiola, Grégoire Michoud, Stilianos Fodelianakis, Ioannis Karakassis, Georgios Kotoulas, Alexandra Pavlidou, Christina Pavloudi, Paraskevi Pitta, Nomiki Simboura, Daniele Daffonchio, Manolis Tsapakis

**Affiliations:** 1 Institute of Oceanography, Hellenic Centre for Marine Research, 71003 Heraklion Crete, Greece; vpitta@hcmr.gr (P.P.); tsapakis@hcmr.gr (M.T.); 2 Department of Biology, University of Crete, 70013 Heraklion Crete, Greece; karakassis@biology.uoc.gr; 3 Institute of Marine Biology, Biotechnology & Aquaculture, 71003 Heraklion Crete, Greece; kotoulas@hcmr.gr (G.K.); cpavloud@hcmr.gr (C.P.); 4 King Abdullah University of Science and Technology, Biological and Environmental Sciences and Engineering Division (BESE), Thuwal 23955-6900, Saudi Arabia; gregoire.michoud@kaust.edu.sa (G.M.); stylianos.fodelianakis@epfl.ch (S.F.); daniele.daffonchio@kaust.edu.sa (D.D.); 5 Institute of Oceanography, Hellenic Centre for Marine Research, 19013 Anavyssos Attiki, Greece; aleka@hcmr.gr (A.P.); msim@hcmr.gr (N.S.)

**Keywords:** community composition, pollution, coastal zone, metagenomics, auxiliary metabolic genes

## Abstract

Viruses interfere with their host’s metabolism through the expression of auxiliary metabolic genes (AMGs) that, until now, are mostly studied under large physicochemical gradients. Here, we focus on coastal marine ecosystems and we sequence the viral metagenome (virome) of samples with discrete levels of human-driven disturbances. We aim to describe the relevance of viromics with respect to ecological quality status, defined by the classic seawater trophic index (TRIX). Neither viral (family level) nor bacterial (family level, based on 16S rRNA sequencing) community structure correlated with TRIX. AMGs involved in the Calvin and tricarboxylic acid cycles were found at stations with poor ecological quality, supporting viral lysis by modifying the host’s energy supply. AMGs involved in “non-traditional” energy-production pathways (3HP, sulfur oxidation) were found irrespective of ecological quality, highlighting the importance of recognizing the prevalent metabolic paths and their intermediate byproducts. Various AMGs explained the variability between stations with poor vs. good ecological quality. Our study confirms the pivotal role of the virome content in ecosystem functioning, acting as a “pool” of available functions that may be transferred to the hosts. Further, it suggests that AMGs could be used as an ultra-sensitive metric of energy-production pathways with relevance in the vulnerable coastal zone and its ecological quality.

## 1. Introduction

Coastal marine ecosystems are frequently under the intense pressure of a wide range of anthropogenic activities, despite their high productivity and provision of goods and services with great socioeconomic imprint [1,2]. Indeed, alterations of ecosystem services have been reported due to enrichment with nutrients and organic matter, chemical and biological pollution (i.e., intrusion of heavy metals, hormones, and antibiotics), and other disturbances, which altogether raise concerns about coastal ecosystem sustainability [2].

Within this context, studying the structure of microbial plankton has recently emerged as a complementary approach to characterize ecosystem status (e.g., [3,4]). Microorganisms are sensitive to low concentrations of contaminants and capable of rapidly responding to environmental perturbations due to their fast growth rates [5,6]. More importantly, microbial, and particularly prokaryotic, communities drive numerous biogeochemical processes in the oceans that determine the cycling of the most important chemical elements [7]. Thus, marine ecosystem conservation and sustainability are reasonably and tightly linked to prokaryotic diversity and function.

While it is nowadays recognized that prokaryotes (hereafter, bacteria) rely upon viruses in multiple ways [8], it is remarkable that the viral community has been barely studied in relation to anthropogenic pressure [9,10]. Viruses modulate microbial community dynamics through mortality, through the alteration of biogeochemical cycling after lysis events, and through the release of labile dissolved organic carbon [11,12]. They are also a driving force in their host’s evolution because they mediate the horizontal transfer of genes, as well as via a range of co-evolutionary processes between hosts and viruses during infection [13]. Lastly, viruses interfere with their host’s metabolism, as they possess genes that, upon their expression, enable them to participate in and redirect the host’s photosynthesis; carbon, sulfur, and fatty-acid metabolism; nutrient acquisition; motility; stress-response; antioxidation; and other processes [14,15]. The discovery of the redirection of the host’s metabolism through the acquisition and expression of these particular viral genes (called auxiliary metabolic genes; AMGs) revolutionized virology and revealed the hidden potential of viruses in the oceanic food web. For instance, through the expression of AMGs, viruses manipulate the cyanobacterial carbon metabolism machinery, aiding in increased viral particle production and ultimate lysis of the host [15]. Moreover, the possession and maintenance of fatty-acid metabolism AMGs in cyanophages offers a fitness advantage toward the modulation of the fluidity of the host cell cytoplasmic or thylakoid membranes [16].

Here, our aim is to examine the whole viral metagenome (virome) in direct relation to human-driven chemical alterations in the coastal zone. More specifically, we attempt to detect differences in viral diversity and AMG content that reflect overall good vs. poor ecological quality. Based on AMG composition, we aim to assess the potential role of viruses in regulating critical host functions under continuously changing conditions in polluted sites. We chose to detect these differences along the Greek coastline under the European Water Framework Directive (WFD, EC 2000) for two reasons: firstly, because the coastline belongs to the top-priority regions for conservation planning in the Mediterranean Sea [17,18,19]. Secondly, because major types of anthropogenic pressure occur there, i.e., aquaculture, agriculture, eutrophication, tourism, dredging, port activity, as well as industry, sewage, and spoil discharges at various intensities [20,21], thus resembling other vulnerable coastal regions worldwide. We assess bacterial taxonomy based on 16S rRNA gene sequencing and viral taxonomy based on virome sequencing. We test the hypothesis that viral community composition and AMG content differ according to the ecological quality of the ecosystem. For this reason, we use the ecological quality trophic index (TRIX) to categorize stations based on their trophic status, which is affected by human-related activities (examples mentioned above). The TRIX is a frequently used index in management assays in the coastal zone [22] that has been optimized for the oligotrophic Eastern Mediterranean Sea [23]. Then, we study the bacterial community composition versus the viral one to find out whether their relation with the TRIX is similar or not. Finally, we present characteristics of the virome that can be used for the evaluation of the ecological status of the coastal zone. While being a totally new and black-box approach for ecosystem management, the study of a virome with its peculiar AMG content may give insights into particular host metabolism pathways that are redirected by viruses in polluted sites.

## 2. Materials and Methods

### 2.1. Sampling Design and Sample-Grouping Substantiation

**Figure 1:**
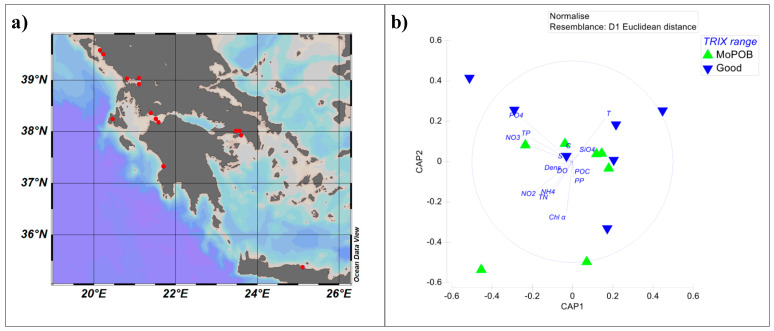
(**a**) Map of the sampling stations in the Ionian and South Aegean seas. (**b**) Sample-grouping substantiation. Canonical analysis of principal coordinates (CAP) of the coastal seawater physicochemical variables (solid-lined circle), depicting the sample grouping based on the ecological quality and Pearson correlations of the physicochemical variables with the canonical axes. SiO4: silicate concentration, DO: dissolved oxygen concentration, POC: particulate organic carbon concentration, TN: total nitrogen concentration, TP: total phosphorus concentration, Chl α: chlorophyll α concentration, PO4: phosphate concentration, NO3 and NO2: nitrate and nitrite concentrations, NH4: ammonium concentration, Dens: density, C: conductivity, S: salinity, T: temperature. Stations are colored based on the annual surface trophic index (TRIX) range and named after “Good” (blue) when having overall good ecological quality (TRIX = 1.6–2.8), and “MoPoB” (green) when having overall poor ecological quality (TRIX > 2.8).

Surface seawater (2 m) was collected from 14 coastal stations in the Ionian and South Aegean seas (Supplementary Information, Table S1, Figure 1a) within the monitoring project European Water Framework Directive 2000/60/EC (WFD) in March 2014 and 2015. Samples were collected by Niskin-bottle deployment on board the R/V Philia and filtered for bacterial 16S rRNA sequencing and viromics. Briefly, 20 L of seawater was collected in acid-cleaned and deionized water-rinsed low-density polyethylene containers and filtered firstly through a 5 μm nylon mesh to remove large particles and then through 0.2 μm polycarbonate filters for bacterial cells, using a Masterflex peristaltic pump under low vacuum (<150 mmHg). The 0.2 μm filtrate was collected in a new container, chemically treated with 1 mg L^−1^ FeCl_3_ to achieve viral particle flocculation within the following 6–12 h [24] and filtered again through 1 μm polycarbonate filters. The 0.2 μm filters were flash-frozen and the 1 μm filters were stored at 4 °C, pending DNA extraction.

Samples were grouped according to the yearly estimated trophic index (TRIX) of the surface waters (2 m), using the formula described in [23] that considers the concentrations of chlorophyll a, dissolved nitrate, nitrite, phosphate, and oxygen in the water column [23]. The detailed data used for the calculation of the annual TRIX are presented in Table S2. Five sampling campaigns (March 2014, September 2014, December 2014, March 2015, and July 2015) were considered, leading to the detection of four levels of ecological quality based on TRIX range: Good (1.6–2.8), Moderate (2.8–4.0), Poor (4.0–5.3), and Bad (>5.3). Stations with Moderate–Poor–Bad ecological quality were grouped together, since the number of stations was too low to support their individual testing. 

To statistically substantiate the grouping of the particular sampling stations based on the annual surface TRIX into “Good” versus “Moderate–Poor–Bad,” we tested their similarities on physicochemical properties, using only the measurements of the specific sampling campaign (2 m depth, March either 2014 or 2015, Table 1). Our goal was to use the annual surface TRIX categorization only if samples were significantly different based on this factor on the March campaigns. The following physicochemical parameters were pre-treated (normalized) in order to compare them under a common scale, and then the normalized matrix was used to create a resemblance matrix using Euclidean distances: temperature, salinity, conductivity, density, and the concentrations of chlorophyll α, dissolved oxygen, nitrate, nitrate, silicate, phosphate, ammonium, total nitrogen, total phosphorus, and particulate organic carbon. Two clusters of samples were defined based on canonical analysis of principal coordinates (CAP) and the clustering was confirmed by PERMANOVA (Pseudo-F_1,12_ = 2.65, *p* < 0.05, Figure 1b). Thus, annual surface TRIX categorization was considered valid for this study. The clusters reflected overall good vs. overall moderate/poor/bad ecological quality (TRIX 1.6–2.8 and TRIX >2.8, respectively). The two levels of the factor “annual surface TRIX” were named after the TRIX range: “Good” and “MoPoB”.

### 2.2. Determination of Physicochemical and Biological Parameters

A conductivity-temperature-depth (CTD) profiler was deployed (Sea-Bird SBE19) to measure temperature and conductivity and to further calculate salinity and density. The concentration of chlorophyll was determined fluorometrically based on [25] (Supplementary Information). The samples for the determination of nitrate, nitrite, silicate, phosphate, ammonium, total phosphorus (TP), and total nitrogen (TN) levels were kept deep frozen (−20 °C) until their analysis in the certified according to ELOT EN ISO/IEC 17025 biogeochemical laboratories of HCMR. Protocols for the above-mentioned nutrients as well as the calculation of particulate organic carbon and particulate phosphorus concentration are described in the Supplementary Information. The abundance of virus-like particles (VLP) was determined based on [26] and the abundance of heterotrophic and autotrophic bacteria was based on [27] from fixed samples with 0.2 μm filtered glutaraldehyde, as described in [28] with slight modifications (Supplementary Information). 

### 2.3. DNA Extraction, Amplification, Sequencing, and Sequence Processing

Viral dsDNA metagenome preparation started by firstly resuspending the 1μm polycarbonate filters in an ascorbic acid buffer [24]. Subsequently, viral and bacterial DNA extraction was done with a cetyl trimethylammonium bromide (CTAB) protocol [29] (Supplementary Information). For the amplification of 16S rRNA gene, polymerase chain reactions were carried out using the locus-specific primers (341f: 5′-CCTACGGGNGGCWGCAG-3′ and 806RB: 5′- GGACTACNVGGGTWTCTAAT -3′, [30,31]) and a universal 5′ tail specified by Illumina (Supplementary Information). Virome reads in FASTQ format were treated as in [32] with modifications in the assembled-contig analysis. The assembled viral contigs were deposited on the zenodo server (doi: 10.5281/zenodo.3929111), and both 16S data and whole viromes were deposited at the National Center for Biotechnology Information (NCBI) BioSample database (accession numbers can be found in Table S1). We used the iVirus pipeline [33] through the Cyverse platform [34] as well as VirFinder [35]: only viral sequences with a VirFinder score ≥0.7 and *p* < 0.05 [35] and VirSorter categories 1 and 2 were used for further analysis [33]. VirSorter provided two types of viral-gene groups: lytic and lysogenic, with both lytic and lysogenic genes being further divided in confidence categories (the most confident ones: category 1 and 2 for lytic and category 5 and 6 for lysogenic, [36]), while firstly eliminating all bacterial genes. The taxonomy of the viral sequences obtained by the VirFinder software was determined by blasting them against the Global Ocean Viromes 2.0 database [37], while the vContact software [38] was used to perform guilt-by-contig-association automatic classification of viral contigs (self-assigned taxonomy of viral sequences). The viral community was determined by counting the occurrence of the corresponding taxa.

Normalization of AMG copy numbers was done using the viral-abundance results of flow cytometry. Firstly, a normalization factor (Table S5) was calculated for each sampling station, by dividing the abundance of total virus-like particles in each station, with the minimum abundance of total virus-like particles of all stations. Then, the normalization factor was multiplied with the AMG abundances. 

Shannon–Wiener’s diversity index was calculated using the PRIMER DIVERSE routine [39,40]. The 16S rRNA gene sequences were quality checked and analyzed using both UPARSE v8 [41] and QIIME v1.9 [42] as described in [32].

### 2.4. Statistics 

Principal coordinates analysis was applied to coordinate the data [43]. Significant differences in the viral and bacterial community patterns of the stations were tested by applying permutational multivariate analysis of variance using the factor “annual surface TRIX” determined by the trophic status of the ecosystem and the resulting TRIX index (as substantiated above). Our null hypothesis was that there are no differences. Bray–Curtis dissimilarity matrices on square-root transformed biological data were constructed [39]. Hypothesis testing was performed using 999 permutations and pairwise tests with a significance level of 0.05. Exploratory analyses were done with the software package PRIMER v6 (PRIMER-E Ltd., Plymouth Marine Laboratory, Natural Environmental Research Council, UK) with PERMANOVA + add-on software [44,45]. Redundancy analysis (RDA) was used to summarize the variation in the biological data (specific viral and bacterial genes’ percentage contribution to total gene copies) that was explained by the abiotic data. The results of this method are presented in RDA plots. Regression analysis was applied to find significant correlations between the total abundance of auxiliary metabolic genes and physicochemical variables. One-way analysis of variance (ANOVA) was applied to find significant differences in the abundance of individual genes between the stations with different ecological qualities (TRIX range). The significance of the differences was assessed with post hoc Tukey test (Tukey HSD). Homogeneity of variance was checked using Levene’s test. Regression and ANOVAs were performed using IBM SPSS statistics software v23.

## 3. Results

### 3.1. Viral Community Composition Does Not Differ Based on Ecological Quality (TRIX Range)

**Figure 2:**
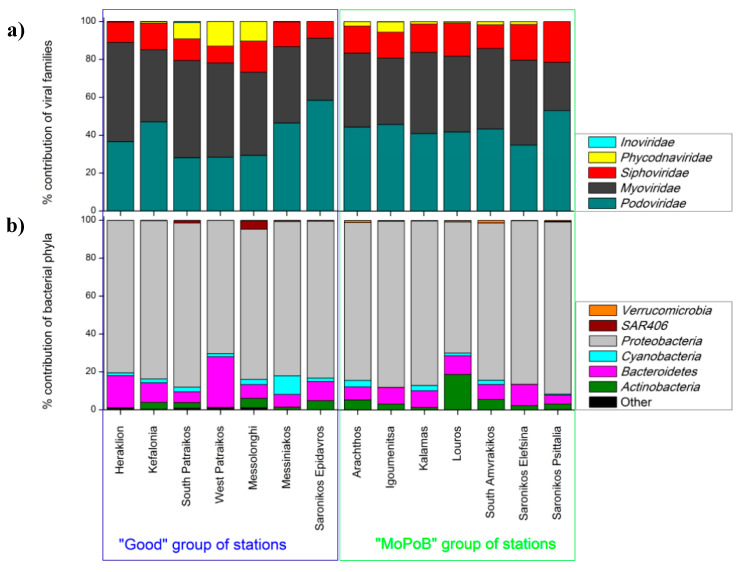
Viral and bacterial community profiles. (**a**) Viral community composition at the family level. (**b**) Bacterial community composition at the phylum level. “Other” bacterial phyla include: *Acidobacteria*, *Armatimonadetes*, *Chlamydiae*, *Chlorobi*, *Chloroflexi*, *Firmicutes*, *Fusobacteria*, *GN02*, *Gemmatimonadetes*, *Kazan-3B-2B*, *Lentisphaerae*, *Nitrospirae*, *OD1*, *OP11*, *OP3*, *SAR406*, *SBR1093*, *SR1*, *Spirochaetes*, *TM6*, *TM7*, *Tenericutes*, *Verrucomicrobia*, *WPS-2*, *WS3*, and *ZB3*. Samples are divided in two groups based on the ecological quality, i.e. the annual TRIX range (left: stations having Good ecological quality, and right: stations having Moderate–Poor–Bad ecological quality).

Table S3 summarizes the raw numbers of viral metagenome reads, the quality of reads, the number of total contigs, the contig length (total, minimum, and maximum), and the N50 contig length. The number of detected viral lytic contigs in our viromes averaged 3386 ± 2534 (Table 2). Within the lytic contigs, only 20% ± 12% were assigned to the family level, agreeing with other deep-sequencing virome surveys regarding the large fraction of unknown viral sequences ([37,46] and references therein). Viral community composition at the family level did not differ based on the seawater ecological quality, which was determined by the TRIX range (PERMANOVA, Pseudo-F_1,12_ = 1.76, *p* > 0.05). Irrespective of ecological quality, *Podoviridae* (43% ± 10% of the assigned contigs, Figure 2a) was as abundant as *Myoviridae* (40% ± 10% of the assigned contigs, Figure 2a), despite the fact that *Myoviridae* usually dominates the virioplankton community on a global scale [47,48]. *Siphoviridae* constituted between 9% and 21% of the assigned contigs (Figure 2a). The highest contribution of *Siphoviridae* was seen in the Saronikos gulf (2 stations) where industrial activity is the largest source of pressure on the total Greek coastline, and this outcome resembled that of other polluted areas too [9]. Evaluation of viral taxonomy at the genus level using a larger set of contigs than the present one would be more suitable for the assessment of viral population variations with respect to ecological quality, since the family level may lead to the wrong impression that the community is even. While the taxonomy assignment using VirSorter provided us with viral genera abundances, we did not use these numbers since the copies of each genus were frequently too low (<100 except for *T4*-like viruses that were detected in higher abundances).

Minor taxonomic differences were detected in two “rare” viral families: a higher percentage contribution of *Phycodnaviridae* was reported at Good stations reaching on average 5% ± 2% of the assigned reads compared to MoPoB, which was 2% ± 2% (Figure 2a, Supplementary Information, Table S4). *Phycodnaviridae* detection was confirmed by the flow cytometry enumeration of high DNA virus-like particles (VLPs) that mostly infect eukaryotic phytoplankton [49]. Indeed, significantly higher contribution of high DNA VLPs was seen at Good (on average >4% of the total VLPs) compared to MoPoB (mostly <3% of the total VLPs, Table 2) as supported by ANOVA (Tukey HSD, *p* < 0.05). The detection of high DNA VLPs and Phycodnaviridae suggests the presence of eukaryotic algae in the Good group of stations [50], which was not apparent based on Chl α data (Table 1).

Apart from *Phycodnaviridae*, the biotechnologically and ecologically important *Inoviridae* family was also not broadly distributed; it reached up to 0.5% of the total assigned reads (Figure 2a, Table S4) at 2 stations within the Good group with mild pressure from sewage pollutants (Kefalonia and South Patraikos). In accordance with our results, the importance of *Inoviridae* in regions that received raw sewage inputs was also noticed elsewhere [9]. The presence of *Inoviridae* in stations with good overall ecological quality may imply the presence of human bacterial pathogens [51,52] in coastal ecosystems, with direct impacts on human health. Assignment to the genera level would enable us to draw safer conclusions about the relation between viral taxonomy and ecological quality.

**Figure 3:**
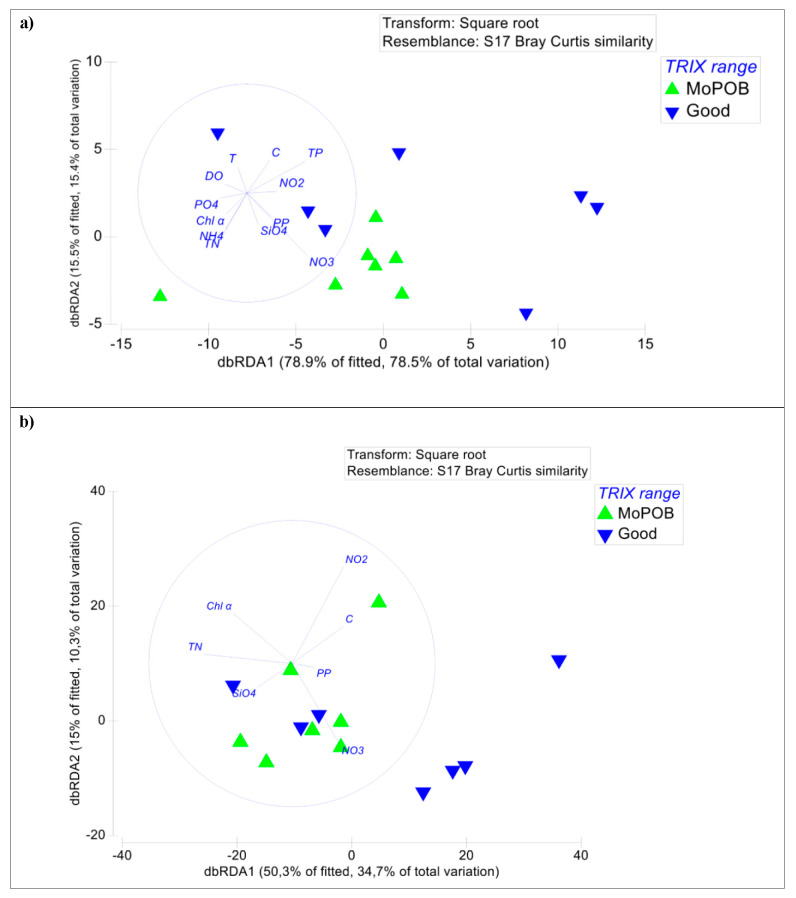
Distance-based redundancy analysis (dbRDA) plot of the distance-based linear model of the physicochemical variables fitted to (**a**) the viral community composition (family level) and (**b**) the bacterial community composition (family level). Samples are presented based on the ecological quality (annual surface TRIX range). The length and direction of the vectors indicate their relative strength and direction of relationship in the ordination plot, respectively. Community similarity was calculated via the Bray–Curtis index after a square–root transformation of the bacterial families read counts. Good: stations having good overall ecological quality and MoPoB: stations having poor and bad overall ecological quality. Selection procedure of variables: forward (Table S7).

A suite of abiotic variables (Figure 3a) explained most of the viral community composition variations (distance-based linear model, adjusted R^2^ = 0.99, selection procedure: forward), with the concentration of NO_3_ being the most important (28.5% of variation, *p* < 0.05). In our study, the average concentrations of NO_3_ as well as other nitrogen forms (NO_2_, NH_4_, TN) and particulate organic carbon concentration (POC) increased with decreasing ecological quality (Table 1). However, the variation in other abiotic parameters within the group of MoPoB (i.e., temperature, salinity, density, SiO_4_, and POC concentrations) did not allow us to draw a firm conclusion about the relation between nutrients and viral community patterns in the examined coastal sites. In other words, while the relation detected here between the viral community and dissolved NO_3_ was surely expected considering previous macro-diversity studies that revealed the strong dependence of the viral community patterns on the environment [37,53], we would need a larger dataset to validate this finding in the coastal zone too.

The need for such validation is particularly important considering that the input of dissolved nutrients at the impacted sites is intense, but frequently the detected concentrations of nitrogen and phosphorus are quite low, reaching oligotrophic levels (e.g., at Amvrakikos gulf). This paradox may indicate (1) fast consumption (2) fast integration into particulate forms and/or (3) removal toward deeper layers in the water column for the dissolved nutrients. These processes have been reported for other regions that receive large inputs of nutrients such as aquaculture farm zones [54,55]. The latter authors described the “disappearance” of added dissolved nutrients released near farms due to their rapid assimilation in the food web. As a result, the relations between gene copies, community structure, and environmental factors should be treated with caution. Future studies should focus on different water layers and other forms of dissolved nutrients to validate the correlations produced in similar datasets. As a matter of fact, a different view of the ecological quality of the stations would be derived in several cases if using the depth-integrated TRIX or the TRIX of different months (e.g., Arachthos, Patraikos, Saronikos), ringing the bell for increased replication.

To sum up, we found that viral taxonomy did not differ according to the ecological quality of the sampling stations at the family level, rejecting our first hypothesis. However, it seems absolutely necessary to evaluate the same hypothesis using viral-genera variations that would probably reveal additional differences. At the family level, viral populations seemed to be uniformly distributed, confirming viromics surveys larger than ours, and highlighting the strong connectivity within the oceans [37,56,57,58]. Until now, the majority of studies have focused on large-scale environmental and geographic variability (e.g., temperature, salinity and pressure gradients) and few others on smaller scales (e.g., [59,60]). Future advances in the resolution of viral taxonomy may change this outcome if one considers that the greatest percentage of contigs remained unassigned in this and in other similar studies. Certainly, the strong relation with the abiotic environment needs further investigation since complex ecological processes such as the fast consumption and remineralization of added nutrients in polluted areas does not guarantee a direct link between nutrient levels and community composition.

### 3.2. AMG Diversity Differs Based on Ecological Quality (TRIX Range)

Stations were significantly different in their total AMG content based on seawater ecological quality (PERMANOVA, Pseudo-F_1,11_ = 4.64, *p* < 0.05). The Shannon–Wiener diversity index of AMGs was on average 5.13 ± 0.06 with small variations between the ecological quality groups (Table 2). The number of total AMG copies followed the pattern of POC concentration (Pearson’s correlation, F_1,13_ = 6.97, R^2^ = 0.767, *p* < 0.05); the highest numbers of AMGs were counted along with the highest organic matter loading (Table 1, Amvrakikos gulf, 3 stations with intense riverine inputs). The positive correlation between POC and total AMG copies could be attributed to the frequent lysis and subsequent release of organic matter and AMGs in the seawater. The positive link between POC and bacterial abundances may also lead to the observed relation between POC concentration and AMG copies, since higher occurrence of bacteria would imply higher occurrence of viruses and, thus, more AMGs too. Either explanation is reinforced by the fact that the ratios between virus-like particles and total bacteria (VBRs) were exceptionally high at these 3 stations (109 ± 24) in contrast to the rest of the MoPoB stations, where VBR was much lower (<76, Table 2). The particular AMGs that contributed the most to the significant differences between Good and MoPoB were the genes *ATPsyn*, *CytC*, *rpe*, *queuosine*, *ACC*, *gltA*, and *cpeT* (>3% contribution each, reaching cumulative contribution 37% for the differences) based on the SIMPER test.

**Figure 4:**
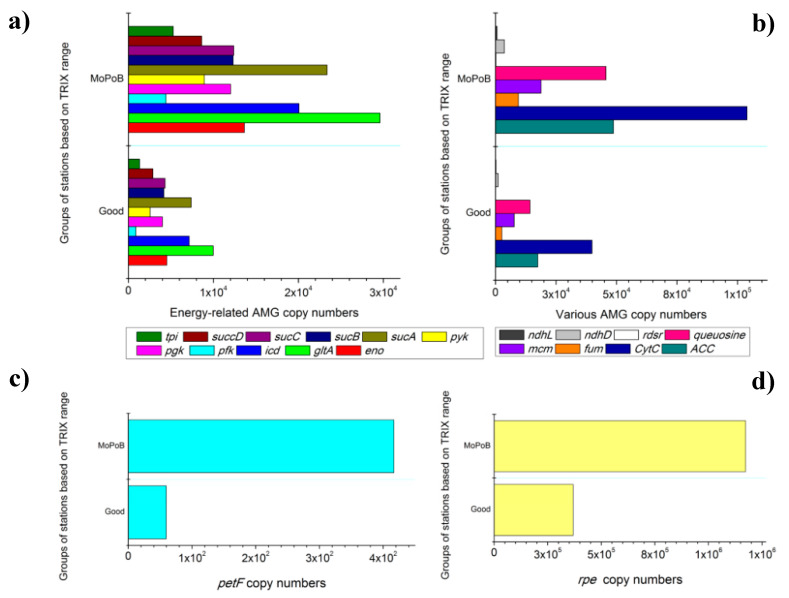
Auxiliary metabolic genes. The normalized abundances of auxiliary metabolic genes that were significantly different between Good and MoPoB group of stations. (**a**) AMGs related to dNTP synthesis and energy production, (**b**): AMGs related to various metabolic processes, (**c**): the *petF* AMG related to photosynthesis, and (**d**): the *rpe* AMG related to energy production. Good: stations having good overall ecological quality and MoPoB: stations having poor overall ecological quality, based on the annual TRIX range.

The potential functional role of AMGs was determined based on earlier reviews [15] and is presented in Table S5. In terms of total AMG copy numbers, the majority of AMGs involved in dNTP synthesis and energy production were significantly more abundant at MoPoB than at Good stations (one-way ANOVAs, Tukey HSD, *p* < 0.1, Table S6). In particular, the central carbon metabolism genes *pgk*, *pfk*, *tpi*, and *pyk* (involved in the modification of the Calvin cycle) were significantly more abundant in stations with poor ecological quality (Figure 4a, Tables S5 and S6), while *zwf*, *talC, gpm*, and *gap* also exhibited higher occurrence in the same group (Table S5). Although the *cp12* AMG was high at several occasions (maximum levels at Arachthos and Saronikos Elefsina stations), the latter observation for the rest energy-production AMGs participating in the Calvin cycle inhibition, suggests that a redirection toward accumulation of ATP, NADPH, and Ru5P for subsequent enhanced dNTP synthesis [61] may have occurred at MoPoB. Given the exceptionally high values of VLPs and VBRs in the MoPoB group, it is probable that extensive dNTP synthesis for virion preparation and fast lysis of the hosts is accomplished through the rapid redirection of host carbon metabolism toward the pentose phosphate pathway (PPP). In addition, the exceptionally high copies of *rpe*, *icd*, *eno*, *gltA* and *sucA*, *sucB*, *sucC* and *sucD* (one-way ANOVAs, Tukey HSD, *p* < 0.1, Figure 4b,d, Tables S5 and S6) may have supported in parallel alternative energy production and dNTP synthesis pathways via the tricarboxylic acid (TCA) cycle. The over-representation of TCA components in stations with overall poor ecological quality (MoPoB) highlight that energy production may be also accomplished by mimicking nutrient-starvation conditions, as recently proposed [62].

AMGs involved in both cycles (Calvin cycle and TCA) eventually support viral lysis by modifying the energy supply to the host. It is noteworthy though that the genes involved in the TCA cycle were exceptionally abundant in the Amvrakikos gulf (3 stations). This gulf receives large amounts of dissolved nutrients due to riverine biogenic inputs and human activities (agriculture and mariculture), which would not support this “starvation” response. However, the fast consumption of the added nutrients and their fast integration into particulate forms (assimilation in the food web), as well as their removal to the deep may lead to the unexpectedly low levels of dissolved nutrients (Table 1) and, further, to the mimicking of starvation responses for energy production. In accordance with this early proposition [62], the highest number of copies of the *rpe* gene was found at the South Amvrakikos gulf (Figure 4d), i.e., at the station with minimum nitrate, nitrite, and phosphate concentrations (Table 1). 

“Non-traditional” energy production pathways were also suspected for the MoPoB group; the 3-hydroxypropionate (3HP) cycle AMGs *acc*, *mcm*, and *fum* were significantly higher at MoPoB than at Good (one-way ANOVA, Tukey HSD, *p* < 0.1, Figure 4b), and the fatty-acid metabolism AMGs *fadb* and *fadL* also exhibited higher occurrence (Table S5). The prevalence of the former 3HP AMGs indicated the redirection toward energy production in the photic zone [15,62] but the exact role of these AMGs in viral genomes is still rather unclear. The highest VBRs were measured at all MoPoB stations (Table 2), indicating rapid infection–lysis cycles that were fueled and sustained by carbon-flow redirection mechanisms. Thus, the input of dissolved nutrients and particulate matter in the human-impacted zone may be accompanied by their rapid turnover via viral lysis.

The abundance of one photosynthetic AMG also increased with increasing overall human pressure (Figure 4c). The electron transfer gene *petF*, encoding for ferredoxin, was more abundant at MoPoB than at Good stations (one-way ANOVAs, Tukey HSD, *p* < 0.1, Table S6). It seems that under poor ecological quality conditions, viruses may respond to balance the redox state of the photosynthetic electron transport chain [63], as *petF* is known to prevent the damage of the photosynthetic reaction centers [64] and to donate electrons to the alternative terminal electron–acceptor cytochrome oxidase [65]. Cyanobacterial damage may have occurred at MoPoB and particularly in areas where industrial activity was the most intense pressure source (Saronikos gulf, 2 stations). The nearly zero occurrence of autotrophic bacteria at the Saronikos gulf (<2 × 10^3^ cells mL^−1^, Table 2) may depict a fraction of this damage too.

Genes from the NDH-1 complex (*ndhD* and *ndhL*, Figure 4b, Table S5) were also significantly more abundant at MoPoB than at Good stations (one-way ANOVA, Tukey HSD, *p* < 0.1, Table S6); possibly aiding photosynthesis. 

Finally, the *rdrs* gene that has been retrieved in metagenomes from oxygen minimum zones was found at all stations; *rdrs* was significantly more abundant in the MoPoB group than in the Good group (one-way ANOVA, Tukey HSD, *p* < 0.05, Figure 4b, Tables S5 and S6). Its involvement in sulfur oxidation and sulfur-based metabolism highlights the potential transfer of these functions to other environments than those detected (e.g., sediment). The consequences of such functional transfer via AMGs between discrete environments were also pointed out previously [57,66]. 

Irrespective of the main metabolic pathways in the community, similar abundances of viruses and bacteria and similar VBR levels may be reached. However, the balance between the Calvin, TCA, and 3HP cycles and the fatty acid and sulfur exploitation determines the quantity and quality of intermediate byproducts. As a result, it is important to study this balance in order to understand community diversity and ecosystem functioning and find the possible connection with the AMG genetic reservoir.

### 3.3. Ecosystem Function Based on Viral and Bacterial Community Composition versus Virome Content

The final goal of this work was to assess the level of ecosystem understanding based on viral and bacterial community composition as well as by the virome content. We tested whether the patterns of bacterial community composition significantly correlated with the trophic index (TRIX), similarly to the test for the viral community presented above. Bacterial community composition at the genus and family levels did not differ between stations based on ecological quality (TRIX range) (PERMANOVA, *p* > 0.05). At the phylum level, significant differences were found between the two groups of stations (PERMANOVA, Pseudo-F_1,12_ = 2.38, *p* < 0.05). The abiotic variables that explained bacterial community patterns at this level were the concentrations of NO_2_, NO_3_, NH_4_ and SiO_4_ as well as water conductivity (distance-based linear model, adjusted R^2^ = 0.56, selection procedure: forward), while at the family level the variables were the same and in addition, the concentrations of Chl α, total nitrogen, and particulate phosphorus (distance-based linear model, adjusted R^2^ = 0.32, selection procedure: forward, Figure 3b, Table S7). Neither viral nor bacterial diversity patterns were reflected on the trophic status of the ecosystem and the resulting ecological quality (TRIX range), rejecting the second hypothesis too. While the use of the bacterial phylum level supports the correspondence to the ecological quality, it also masks major community differences between the tested stations.

Shannon–Wiener diversity based on the bacterial operation-taxonomic units (out) data was on average 5.11 ± 0.60 (family level), with most of the lowest values found at MoPoB (Table 2). The dominant phylum was *Proteobacteria* with a significantly higher percentage contribution at MoPoB (84% ± 7%) compared to Good (81% ± 5%), followed by a similar pattern of *Actinobacteria*: 6% ± 6% and 3% ± 2% at MoPoB and Good, respectively (Figure 2b). *Actinobacteria*’s higher contribution at MoPoB may indicate the presence of complex organic matter and xenobiotics at regions that receive high amounts of nutrients, pollutants, and waste [67]. On the contrary, *Cyanobacteria* contributed more at Good (3% ± 3%) than MoPoB (1% ± 1%, Figure 2b), confirming the flow cytometry enumeration of cyanobacteria (Table 2). On the other hand, *Bacteroidetes* showed a consistently high percentage contribution at MoPoB (8% ± 2%) but a highly variable one at Good (12% ± 7%, Figure 2b). Rare phyla contributed up to 1% at few stations within the Good group (West Patraikos and Messolonghi), while especially Messolonghi was also characterized by a high percentage contribution of *SAR406* (4.5% of the total reads). 

Bacterial taxa indicative of pollution and, more specifically, of raw anthropogenic inputs [68] were found at all stations. For instance, members of the families *Caulobacteraceae*, *Oxalobacteraceae*, *Hyphomicrobiaceae*, *Microbacteriaceae*, *Saprospirae*, *SAR324*, *Methylophilaceae*, and *Thiohalorhabdales* were detected at low percentage contributions throughout the coastal zone, indicating the widespread presence of sewage-related and heavy-metal-tolerant taxa. Several pathogens were also detected at all stations, including *Streptococcus*, *Lactobacillus*, *Campylobacterales*, *HTCC2188*, *HTCC2207*, *Bacteroides*, *Clostridia*, *Enterobacteriaceae*, and *Dechloromonas* [69]. 

Our study confirmed that bacterial as well as viral community structure does not depict the ecosystem’s ecological status based on classic ecological indices such as the TRIX. This lack of dependence between community composition and trophic status (i.e., dissolved nutrient and oxygen concentration) may lead to improper management of polluted regions. In fact, newly developed ecosystem-management tools consider the microbial community composition [4] and research invests heavily in this direction. However, early signals of alternative energy production pathways can be revealed through viromics (e.g., sulfur-based, 3HP-based, and fatty-acid based metabolism); this was the case at sites with good overall ecological status. Certainly, the study of the virome is by far more expensive than the 16S-based bacterial community assessment and, thus, less suitable for mainstream management assays. In addition, while the whole virome can nowadays support viral taxonomic assessment, a cheaper PCR-based assay would not be appropriate since there is no universal gene for the viral community such as the 16S rRNA for the bacterial community. Similarly, the taxonomic affiliation of AMGs is currently rather limited (studied either via the whole metagenome or a PCR-based approach). Finally, viromics should be accompanied by certain measurements to aid an ecological perspective; namely, transcriptomics analysis would ensure that AMGs are indeed expressed in the system, while the bacterial metagenome should also be tested for the occurrence of the same genes. What we know so far is that AMGs do not only “occur” in the ecosystem but actually depict its functional potential, as they are expressed under appropriate environmental conditions. The occurrence of several AMGs under good overall ecological quality is an indication that fitness advantages are transferred between hosts and viruses, and as a result, the whole trophic web function and diversity evolves and creates different ecological niches than what would exist without the contribution of these genes.

Apart from AMGs and their functional potential, the virome holds information about the occurrence of lysogeny either through the taxonomic assignment of lysogenic genes (e.g., with VirSorter, [33]) or through the detection of lysogeny-related genes (e.g., integrase, plasmid partition genes, [70]). Here, the number of lysogenic contigs based on the most confident category was not adequate to allow for statistical evaluation of their variation and taxonomy. Notwithstanding this, it is noteworthy that a few *attP* copies were putatively assigned to bacterial hosts based on exact matches with the bacterial *attB* (Table S8); Spirochaetes and Synergistetes bacterial phyla were only found based on this search (not based on 16S rRNA gene assignment), highlighting the potential occurrence of anaerobic conditions, sulfur metabolism, oil pollution as well as the presence of human pathogens [71,72,73] at the Good group of stations.

## 4. Conclusions

Nowadays, one of the key questions in marine ecosystem monitoring and conservation is which biological component(s) should be monitored for the detection and management of pollution. As Nõges *et al.* [74] recently pointed out, such information usually derives from insensitive, thus inappropriate taxa. In our study we focus on viruses, which are basic components of aquatic and terrestrial food webs. We highlight that the virome contains ecologically meaningful information about the host metabolic processes that are manipulated by viruses and we describe their relation with the ambient environment. Specifically, the viral auxiliary metabolic genes—either expressed or “simply” carried in the community—play a pivotal role in ecosystem functioning: AMGs can be integrated in the host genome and transferred as lysogenic to next host generations, subsequently transferring their function too. In addition, although the infected host cell eventually bursts, AMGs can instantly create the appropriate conditions to overcome bottlenecks for host production.

We detected genes involved in redox balance, sulfur-, and fatty acid- based metabolism as well as anaerobic metabolism in areas that would be characterized as having good ecological status based on classic indices (here, the TRIX). Although we do not provide information on actual viral gene expression, the potential for host-metabolism redirection remains at a dormant-like state within the available pool of viral functional genes. This pool can act as an alternative ultra-sensitive metric of energy-production pathways (e.g., TCA vs. fatty-acid-based metabolism) in vulnerable ecosystems where the physicochemical conditions change rapidly. As discussed above, such an approach requires further investigation with a virome dataset larger than the presented one, as well as confirmation of respective bacterial functions with transcriptome analysis and elimination of the possibility to confuse bacterial with viral AMGs. We also found that neither the viral nor the bacterial taxonomy at the family or genus level differed based on the ecological quality of the ecosystem. “Typical” bacterial and viral taxa of polluted sites were found under all levels of ecological quality. Thus, taxonomic differences cannot yet be evaluated in an ecological context, while the diversity of AMGs can uncover the direction of energy production and organic matter circulation. Transcending the era of trying to answer “who does what?,” studying the pool of available metabolic functions within both the viral and host metagenomes may be more meaningful than studying the mere community structure, especially under the scope of the drastic anthropogenic impacts on the marine ecosystem.

## Figures and Tables

**Table 1:** Abiotic variables. Temperature (T, ^o^C), conductivity (C, mS cm^1^), salinity (*S*, psu), density (Dens, kg m^−3^) and the concentrations of dissolved oxygen (DO, mmole L^−1^), DO (% DO Dev), silicate (SiO_4_, μM), phosphate (PO_4_, μg L^−1^), nitrate (NO_3_, μg L^−1^), nitrite (NO_2_, μg L^−1^), ammonium (NH_4_, μg L^−1^), total nitrogen (TN, μM), total phosphorus (TP, μM), particulate phosphorus (PP, μΜ), particulate organic carbon (POC, μM), and chlorophyll α (Chl α, μg L^−1^), along with the resulting TRIX (surface-waters March TRIX values and categories). The annual surface TRIX (surface-waters annual TRIX values and categories) are presented in capital letters and were used for the grouping of the sampling stations in this study. The depth-integrated annual TRIX is also presented in the last column for comparison with the surface ones.

Station ID	T	C	*S*	Dens	DO	DO Dev%	SiO_4_	PO_4_	NO_3_	NO_2_	NH_4_	TN	TP	PP	POC	Chl α	Surface-Waters March TRIX	Surface-Waters Annual TRIX	Depth-Integrated Annual TRIX
Heraklion	17.49	49.91	38.94	28.80	5.30	0.14	0.99	2.85	3.26	0.48	5.34	0.00	0.00	0.06	1.74	0.24	1.19 (H)	2.22 (GOOD)	1.75 (G)
Kefalonia	14.18	45.33	38.01	28.47	5.78	1.75	1.56	2.25	9.67	1.56	3.29	7.73	0.23	0.09	11.58	0.94	2.69 (G)	2.62 (GOOD)	2.87 (M)
South Patraikos	14.76	46.08	38.15	28.46	5.88	4.87	4.87	13.25	350.11	8.65	15.96	8.00	0.80	0.09	14.00	0.43	4.60 (P)	2.12 (GOOD)	2.45 (G)
West Patraikos	14.18	45.78	38.44	28.81	5.88	3.75	4.97	8.30	57.47	7.87	3.29	6.38	0.54	0.06	10.83	0.33	3.63 (M)	2.58 (GOOD)	3.02 (M)
Messolonghi	14.05	45.09	37.91	28.43	5.79	1.50	3.69	2.25	48.79	7.91	3.11	6.40	0.20	0.04	7.02	0.33	2.77 (G)	2.21 (GOOD)	2.06 (G)
Messiniakos	16.37	48.07	38.39	28.28	5.45	0.35	1.49	3.69	13.21	3.17	10.77	4.49	0.10	0.01	5.52	0.21	1.97 (G)	2.19 (GOOD)	2..16 (G)
Saronikos Epidavros	14.73	46.50	38.56	28.78	5.98	6.73	0.56	2.80	0.74	2.35	0.92	6.09	0.16	0.05	11.39	0.40	2.48 (G)	2.14 (GOOD)	5.32 (B)
Arachthos	14.78	36.31	29.23	21.57	6.65	11.74	21.62	4.19	6.70	2.25	0.92	10.53	0.45	0.15	34.65	0.12	2.73 (G)	3.73 (MODERATE)	4.47 (P)
Igoumenitsa	13.81	44.22	37.32	28.02	5.73	0.27	3.02	1.71	49.48	2.67	8.28	8.75	0.30	0.12	15.44	1.10	2.5 (G)	3.83 (MODERATE)	3.82 (M)
Kalamas	15.51	46.24	37.56	27.83	5.61	4.99	4.24	1.64	39.18	5.29	2.87	5.41	0.17	0.04	8.99	0.52	2.88 (M)	3.25 (MODERATE)	3.45 (M)
Louros	15.23	33.83	26.72	19.55	6.79	12.80	23.28	7.26	45.20	3.36	0.92	16.40	0.62	0.18	53.56	0.06	3.30 (M)	4.46 (POOR)	4.39 (P)
South Amvrakikos	14.47	34.73	28.03	20.71	6.68	10.82	24.87	3.17	1.12	2.07	0.92	10.87	0.45	0.13	50.73	0.17	2.41 (G)	3.15 (MODERATE)	5.77 (B)
Saronikos Elefsina	12.72	43.44	37.66	28.51	5.98	2.00	1.79	5.56	281.17	7.54	16.79	16.93	0.29	0.07	17.28	0.64	4.03 (P)	4.22 (POOR)	3.67 (M)
Saronikos Psittalia	14.33	46.13	38.61	28.90	5.51	2.38	1.34	11.61	44.21	5.34	11.52	6.85	0.32	0.05	9.31	0.64	3.78 (M)	3.52 (MODERATE)	3.62 (M)

**Table 2:** Biotic variables. The number of viral lytic contigs detected and the percentage of assigned viral lytic contigs to the taxonomic level of order, family, or genus (% assigned viral lytic). The abundances of autotrophic bacteria (AB, cells mL^−1^), heterotrophic bacteria (HB, cells mL^−1^), virus-like particles (VLP, VLPs mL^−1^) and total bacteria (TB, cells mL^−1^), the ratio between virus-like particles and total bacteria (VBR), and the percentages of low-, medium-, and high-DNA-content VLPs (%LDNA V, %MDNA V, %HDNA V, respectively) and low- and high-DNA-content HB (%LDNA B and %HDNA B, respectively). Shannon–Wiener diversity indices based on 16S-derived operational taxonomic unit table on the family level (S-W Bacteria) and the viral metagenomic assignment of viral families (S-W Viruses). The total gene copies of AMGs and the Shannon–Wiener diversity index based on AMG content (S-W AMGs). Average and standard deviation (stdev) values are presented based on the two ecological quality categories.

Sample ID	Lytic Contigs	% Assigned Viral	AB	HB	VLP	TB	VBR	%LDNA V	%MDNA V	%HDNA V	%LDNA B	%HDNA B	S-W Bacteria	S-W Viruses	Total AMGs	S-W AMGs
Heraklion	638	8.0%	3 × 10^4^	9 × 10^5^	9 × 10^6^	9 × 10^5^	10.69	72.8%	23.4%	3.7%	44.6%	55.4%	5.92	1.67	1,493,947	5.11
Kefalonia	700	25.1%	3 × 10^4^	9 × 10^5^	2 × 10^7^	1 × 10^6^	16.79	78.9%	17.1%	4.0%	58.2%	41.8%	4.82	1.87	636,782	5.15
South Patraikos	185	9.9%	2 × 10^4^	5 × 10^5^	9 × 10^6^	5 × 10^5^	17.65	62.8%	29.5%	7.7%	52.7%	47.3%	6.08	2.07	456,142	5.06
West Patraikos	261	9.1%	1 × 10^4^	4 × 10^5^	8 × 10^6^	4 × 10^5^	17.07	63.5%	27.4%	9.1%	60.4%	39.6%	5.82	1.92	395,112	5.04
Messolonghi	116	17.5%	1 × 10^4^	4 × 10^5^	1 × 10^7^	4 × 10^5^	32.26	75.2%	18.9%	5.9%	62.0%	38.0%	6.03	1.95	164,837	5.12
Messiniakos	521	31.5%	5 × 10^4^	2 × 10^6^	1 × 10^7^	2 × 10^6^	5.04	82.0%	15.6%	2.4%	68.3%	31.7%	4.76	1.67		
Saronikos Epidavros	125	28.1%	2 × 10^4^	8 × 10^5^	1 × 10^7^	9 × 10^5^	16.10	68.7%	26.9%	4.4%	54.8%	45.2%	4.56	1.49	325,742	5.16
Arachthos	805	16.8%	1 × 10^5^	4 × 10^5^	6 × 10^7^	5 × 10^5^	111.33	67.1%	26.9%	6.0%	35.0%	65.0%	4.82	1.83	1,319,746	5.21
Igoumenitsa	1196	16.0%	1 × 10^4^	2 × 10^6^	2 × 10^7^	2 × 10^6^	12.06	76.9%	16.7%	6.4%	63.1%	36.9%	4.34	1.98	1,387,594	5.07
Kalamas	1009	15.3%	5 × 10^4^	1 × 10^6^	1 × 10^7^	1 × 10^6^	13.55	78.1%	18.5%	3.4%	55.3%	44.7%	5.04	1.79	545,599	5.21
Louros	736	17.7%	2 × 10^5^	7 × 10^5^	8 × 10^7^	9 × 10^5^	84.19	78.2%	18.1%	3.7%	36.2%	63.8%	5.13	1.76	4,532,574	5.21
South Amvrakikos	425	9.2%	1 × 10^5^	3 × 10^5^	6 × 10^7^	5 × 10^5^	131.32	75.4%	20.4%	4.3%	36.2%	63.8%	4.78	1.80	3,361,543	5.06
Saronikos Elefsina	1387	38.1%	2 × 10^2^	8 × 10^5^	6 × 10^7^	8 × 10^5^	75.73	79.4%	18.4%	2.2%	30.6%	69.4%	4.55	1.81	1,530,439	5.15
Saronikos Psittalia	98	9.4%	2 × 10^3^	8 × 10^5^	2 × 10^7^	8 × 10^5^	22.71	77.1%	20.4%	2.5%	58.6%	41.4%	4.86	1.56	380,858	5.13

## Supplementary Materials

The following are available online at https://www.mdpi.com/1999-4915/12/8/806/s1, The Supplementary Information file includes Tables S1–S8 as well as methodological details for the determination of physicochemical and biological variables and for DNA extraction, amplification, sequencing and sequence processing protocols.

## References

[1] Nogales B., Lanfranconi M.P., Piña-Villalonga J.M., Bosch R. (2011). Anthropogenic perturbations in marine microbial communities. FEMS Microbiol. Rev..

[2] Halpern B.S., Frazier M., Potapenko J., Casey K.S., Koenig K., Longo C., Lowndes J.S., Rockwood R.C., Selig E.R., Selkoe K.A. (2015). Spatial and temporal changes in cumulative human impacts on the world’s ocean. Nat. Commun..

[3] Quero G.M., Cassin D., Botter M., Perini L., Luna G.M. (2015). Patterns of benthic bacterial diversity in coastal areas contaminated by heavy metals, polycyclic aromatic hydrocarbons (PAHs) and polychlorinated biphenyls (PCBs). Front. Microbiol..

[4] Borja A. (2018). Testing the efficiency of a bacterial community-based index (microgAMBI) to assess distinct impact sources in six locations around the world. Ecol. Indic..

[5] Sun M.Y., Dafforn K.A., Brown M.V., Johnston E.L. (2012). Bacterial communities are sensitive indicators of contaminant stress. Mar. Pollut. Bull..

[6] Ager D., Evans S., Li H., Lilley A.K., Gast C.J. (2010). Van Der Anthropogenic disturbance affects the structure of bacterial communities. Environ. Microbiol..

[7] Falkowski P.G., Fenchel T., Delong E.F. (2008). The microbial engines that drive earth’s biogeochemical cycles. Science (80-.).

[8] Warwick-Dugdale J., Buchholz H.H., Allen M.J., Temperton B. (2019). Host-hijacking and planktonic piracy: How phages command the microbial high seas. Virol. J..

[9] Cantalupo P.G., Calgua B., Zhao G., Hundesa A., Wier A.D., Katz J.P., Grabe M., Hendrix R.W., Girones R., Wang D. (2011). Raw Sewage Harbors Diverse Viral Populations. MBio.

[10] Ng T.F.F., Marine R., Wang C., Simmonds P., Kapusinszky B., Bodhidatta L., Oderinde B.S., Wommack K.E., Delwart E., Phyloge- N. (2012). High Variety of Known and New RNA and DNA Viruses of Diverse Origins in Untreated Sewage. J. Virol..

[11] Suttle C. (2007). A Marine viruses--major players in the global ecosystem. Nat. Rev. Microbiol..

[12] Keshri J., Sriram A., Ram P., Colombet J., Perriere F. (2017). Differential impact of lytic viruses on the taxonomical resolution of freshwater bacterioplankton community structure. Water Res..

[13] Koskella B., Brockhurst M.A. (2014). Bacteria-phage coevolution as a driver of ecological and evolutionary processes in microbial communities. FEMS Microbiol. Ecol..

[14] Brum J.R., Sullivan M.B. (2015). Rising to the challenge: Accelerated pace of discovery transforms marine virology. Nat. Rev. Microbiol..

[15] Hurwitz B.L., U’Ren J.M. (2016). Viral metabolic reprogramming in marine ecosystems. Curr. Opin. Microbiol..

[16] Roitman S., Hornung E., Flores-Uribe J., Sharon I., Feussner I., Beja O., Biology F., Israel T. (2018). Cyanophage-encoded lipid desaturases: Oceanic distribution, diversity and function. ISME J..

[17] Micheli F., Levin N., Giakoumi S., Katsanevakis S., Abdulla A., Coll M., Fraschetti S., Kark S., Koutsoubas D., Mackelworth P. (2013). Setting Priorities for Regional Conservation Planning in the Mediterranean Sea. PLoS ONE.

[18] Stock A., Crowder L.B., Halpern B.S., Micheli F. (2018). Uncertainty analysis and robust areas of high and low modeled human impact on the global oceans. Conserv. Biol..

[19] Piante C., Ody D. (2015). Blue Growth in the Mediterranean Sea: The Challenge of Good Environmental Status.

[20] Simboura N., Pavlidou A., Bald J., Tsapakis M., Pagou K., Zeri C., Androni A., Panayotidis P. (2016). Response of ecological indices to nutrient and chemical contaminant stress factors in Eastern Mediterranean coastal waters. Ecol. Indic..

[21] Simboura N., Maragou P., Paximadis G., Kapiris K., Papadopoulos V., Sakellariou D., Pavlidou A., Hatzianestis I., Salomidi M., Arvanitidis C., Sheppard C. (2019). Ch. 9. Greece. World Seas: An Environmental Evaluation Volume I: Europe, The Americas and West Africa.

[22] Borja A., Prins T.C., Simboura N., Andersen J.H., Berg T., Marques J., Neto J.M., Papadopoulou N., Reker J., Teixeira H. (2014). Tales from a thousand and one ways to integrate marine ecosystem components when assessing the environmenta l status. Front. Mar. Sci..

[23] Primpas I., Karydis M. (2011). Scaling the trophic index (TRIX) in oligotrophic marine environments. Environ. Monit. Assess..

[24] John S.G., Mendez C.B., Deng L., Poulos B., Kauffman A.K.M., Kern S., Brum J., Polz M.F., Boyle E.A., Sullivan M.B. (2011). A simple and efficient method for concentration of ocean viruses by chemical flocculation. Environ. Microbiol. Rep..

[25] Holm-Hansen O., Lorenzen C., Holmes R., Strickland J. (1965). Fluorometric determination of chlorophyll. CIES J. Mar. Sci..

[26] Brussaard C.P.D. (2004). Optimization of Procedures for Counting Viruses by Flow Cytometry. Appl. Environ. Microbiol..

[27] Marie D., Partensky F., Jacquet S., Vaulot D. (1997). Enumeration and cell cycle analysis of natural populations of marine picoplankton by flow cytometry using the nucleic acid stain SYBR Green I. Appl. Environ. Microbiol..

[28] Tsiola A., Pitta P., Giannakourou A., Bourdin G., Marro S., Maugendre L., Pedrotti M.L., Gazeau F. (2017). Ocean acidification and viral replication cycles: Frequency of lytically infected and lysogenic cells during a mesocosm experiment in the NW Mediterranean Sea. Estuar. Coast. Shelf Sci..

[29] Winnepenninckx B.T., Backeljau T., Wachter R. (1993). De Extraction of high molecular weight DNA from molluscs. Trends Genet..

[30] Klindworth A., Pruesse E., Schweer T., Peplies J., Quast C., Horn M., Glöckner F.O. (2013). Evaluation of general 16S ribosomal RNA gene PCR primers for classical and next-generation sequencing-based diversity studies. Nucleic Acids Res..

[31] Apprill A., Mcnally S., Parsons R., Weber L. (2015). Minor revision to V4 region SSU rRNA 806R gene primer greatly increases detection of SAR11 bacterioplankton. Aquat. Microb. Ecol..

[32] Tsiola A., Toncelli C., Fodelianakis S., Michoud G., Bucheli T.D., Gavriilidou A., Kagiorgi M., Kalantzi I., Knauer K., Kotoulas G. (2018). Low-dose addition of silver nanoparticles stresses marine plankton communities. Environ. Sci. Nano.

[33] Bolduc B., Youens-Clark K., Roux S., Hurwitz B.L., Sullivan M.B. (2017). iVirus: Facilitating new insights in viral ecology with software and community data sets imbedded in a cyberinfrastructure. ISME J..

[34] Goff S.A., Vaughn M., McKay S., Lyons E., Stapleton A.E., Gessler D., Matasci N., Wang L., Hanlon M., Lenards A. (2011). The iPlant Collaborative: Cyberinfrastructure for Plant Biology. Front. Plant. Sci..

[35] Ren J., Ahlgren N.A., Lu Y.Y., Fuhrman J.A., Sun F. (2017). VirFinder: A novel k -mer based tool for identifying viral sequences from assembled metagenomic data. Microbiome.

[36] Roux S., Enault F., Hurwitz B.L., Sullivan M.B. (2015). VirSorter: Mining viral signal from microbial genomic data. PeerJ.

[37] Gregory A.C., Zayed A.A., Sunagawa S., Wincker P., Sullivan M.B., Ferland J., Kandels S., Liu Y., Marec C., Vik D. (2019). Marine DNA Viral Macro- and Microdiversity from Pole to Pole Article Marine DNA Viral Macro- and Microdiversity from Pole to Pole. Cell.

[38] Bolduc B., Jang H.B., Doulcier G., You Z., Roux S., Sullivan M.B. (2017). vConTACT: An iVirus tool to classify double-stranded DNA viruses that infect Archaea and Bacteria. PeerJ.

[39] Clarke K.R., Warwick R.M. (1994). Change in Marine Communities: An Approach to Statistical Analysis and Interpretation.

[40] Pielou E. (1971). An Introduction to Mathematical Ecology.

[41] Edgar R.C. (2013). UPARSE: Highly accurate OTU sequences from microbial amplicon reads. Nat. Methods.

[42] Caporaso J.G., Lauber C.L., Walters W.A., Berg-Lyons D., Huntley J., Fierer N., Owens S.M., Betley J., Fraser L., Bauer M. (2012). Ultra-high-throughput microbial community analysis on the Illumina HiSeq and MiSeq platforms. ISME J..

[43] Clarke K.R., Ainsworth M. (1993). A method of linking multivaritate community structure to environmental variable. Mar. Ecol. Prog. Ser..

[44] Anderson M.J., Gorley R.N., Clarke K.R. (2008). PERMANOVA + for PRIMER: Guide to Software and Statistical Methods.

[45] Somerfield P.J. (2008). Identification of the Bray-Curtis similarity index: Comment on Yoshioka (2008). Mar. Ecol. Prog. Ser..

[46] Ruiz-Perez C.A., Tsementzi D., Hatt J.K., Sullivan M.B., Konstantinidis K.T. (2019). Prevalence of viral photosynthesis genes along a freshwater to saltwater transect in Southeast USA. Environ. Microbiol. Rep. Rep..

[47] Yooseph S., Sutton G., Rusch D.B., Halpern A.L., Williamson S.J., Remington K., Eisen J.A., Heidelberg K.B., Manning G., Li W. (2007). The Sorcerer II global ocean sampling expedition: Expanding the universe of protein families. PLoS Biol..

[48] Williamson S.J., Rusch D.B., Yooseph S., Halpern A.L., Heidelberg K.B., Glass J.I., Pfannkoch C.A., Fadrosh D., Miller C.S., Sutton G. (2008). The Corcerer II global ocean sampling expedition: Metagenomic characterization of viruses within aquatic microbial samples. PLoS ONE.

[49] Larsen J.B., Larsen A., Thyrhaug R., Bratbak G., Sandaa R. (2008). A Response of marine viral populations to a nutrient induced phytoplankton bloom at different pCO2 levels. Biogeosciences.

[50] Weynberg K.D., Allen M.J., Ashelford K., Scanlan D.J., Wilson W.H. (2009). From small hosts come big viruses: The complete genome of a second Ostreococcus tauri virus, OtV-1. Environ. Microbiol..

[51] Roux S., Krupovic M., Daly R.A., Borges A.L., Nayfach S., Schulz F., Sharrar A., Carnevali P.B.M., Cheng J., Ivanova N.N. (2019). Cryptic inoviruses revealed as pervasive in bacteria and archaea across Earth’s biomes. Nat. Microbiol..

[52] McMinn B.R., Ashbolt N.J., Korajkic A. (2018). Bacteriophages as indicators of fecal pollution and enteric virus removal. Lett. Appl. Microbiol..

[53] Brum J.R., Ignacio-espinoza J.C., Roux S., Doulcier G., Acinas S.G., Alberti A., Chaffron S., Cruaud C., de Vargas C., Gasol J.M. (2015). Patterns and ecological drivers of ocean viral communities. Science (80-.).

[54] Pitta P., Tsapakis M., Apostolaki E., Tsagaraki T., Holmer M., Karakassis I. (2009). ‘Ghost nutrients’ from fish farms are transferred up the food web by phytoplankton grazers. Mar. Ecol. Prog. Ser..

[55] Tsagaraki T.M., Pitta P., Frangoulis C., Petihakis G., Karakassis I. (2013). Plankton response to nutrient enrichment is maximized at intermediate distances from fish farms. Mar. Ecol. Prog. Ser..

[56] Coutinho F.H., Silveira C.B., Gregoracci G.B., Thompson C.C., Edwards R.A., Brussaard C.P.D., Dutilh B.E., Thompson F.L. (2017). Marine viruses discovered via metagenomics shed light on viral strategies throughout the oceans. Nat. Commun..

[57] Dann L.M., Rosales S., Mckerral J., Paterson J.S., Smith R.J., Jeffries T.C., Oliver R.L., Mitchell J.G. (2016). Marine and giant viruses as indicators of a marine microbial community in a riverine system. Microbiologyopen.

[58] Vellend M., Lajoie G., Bourret A., Murria C., Kembel S.W., Garant D. (2014). Drawing ecological inferences from coincident patterns of population- and community-level biodiversity. Mol. Ecol..

[59] Allen L.Z., Mccrow J.P., Ininbergs K., Dupont C.L., Badger J.H., Hoffman J.M., Ekman M., Allen A.E., Bergman B., Craig J. (2017). The Baltic Sea Virome: Diversity and transcriptional activity of DNA and RNA viruses. mSystems.

[60] Castelán-Sánchez H.G., Lopéz-Rosas I., García-Suastegui W.A., Peralta R., Dobson A.D.W., Batista-García R.A., Dávila-ramos S. (2019). Extremophile deep-sea viral communities from hydrothermal vents: Structural and functional analysis. Mar. Genomics.

[61] Thompson L.R., Zeng Q., Kelly L., Huang K.H., Singer A.U., Stubbe J., Chisholm S.W. (2011). Phage auxiliary metabolic genes and the redirection of cyanobacterial host carbon metabolism. Proc. Natl. Acad. Sci. USA.

[62] Hurwitz B.L., Hallam S.J., Sullivan M.B. (2013). Metabolic reprogramming by viruses in the sunlit and dark ocean. Genome Biol..

[63] Puxty R.J., Millard A.D., Evans D.J., Scanlan D.J. (2015). Shedding new light on viral photosynthesis. Photosynth. Res..

[64] Sullivan M.B., Huang K.H., Ignacio-Espinoza J.C., Berlin A.M., Kelly L., Weigele P.R., DeFrancesco A.S., Kern S.E., Thompson L.R., Young S. (2010). Genomic analysis of oceanic cyanobacterial myoviruses compared with T4-like myoviruses from diverse hosts and environments. Environ. Microbiol..

[65] Gao E., Huang Y., Ning D. (2016). Metabolic Genes within Cyanophage Genomes: Implications for diversity and evolution. Genes.

[66] Roux S., Brum J.R., Dutilh B.E., Sunagawa S., Duhaime M.B., Loy A., Poulos B.T., Solonenko N., Lara E., Poulain J. (2016). Ecogenomics and potential biogeochemical impacts of globally abundant ocean viruses. Nature.

[67] Alvarez A., Saez J.M., Davila Costa J.S., Colin V.L., Fuentes M.S., Cuozzo S.A., Benimeli C.S., Polti M.A., Amoroso M.J. (2017). Actinobacteria: Current research and perspectives for bioremediation of pesticides and heavy metals. Chemosphere.

[68] Zhang S., Tsementzi D., Hatt J.K., Bivins A., Khelurkar N., Brown J. (2019). Intensive allochthonous inputs along the Ganges River and their effect on microbial community composition and dynamics. Environ. Microbiol..

[69] Liu S.G., Luo Y.R., Huang L.F. (2016). Dynamics of size-fractionated bacterial communities during the coastal dispersal of treated municipal effluents. Appl. Microbiol. Biotechnol..

[70] Bellas C.M., Anesio A.M., Barker G. (2015). Analysis of virus genomes from glacial environments reveals novel virus groups with unusual host interactions. Front. Microbiol..

[71] Hampson D.J., Ahmed N. (2009). Spirochaetes as intestinal pathogens: Lessons from a Brachyspira genome. Gut Pathog..

[72] Eggers C.H., Caimano M.J., Malizia R.A., Kariu T., Cusack B., Desrosiers D.C., Hazlett K.R.O., Claiborne A., Pal U., Radolf J.D. (2011). The coenzyme A disulphide reductase of Borrelia burgdorferi is important for rapid growth throughout the enzootic cycle and essential for infection of the mammalian host. Mol. Microbiol..

[73] Jumas-Bilak E., Marchandin H., Rosenberg E., DeLong E.F., Lory S., Stackebrandt E., Thompson F. (2014). The Phylum Synergistetes. The Prokaryotes.

[74] Nõges P., Argillier C., Borja Á., Mikel J., Kode V., Pletterbauer F., Sagouis A., Birk S. (2016). Quantified biotic and abiotic responses to multiple stress in freshwater, marine and ground waters. Sci. Total Environ..

