# Supplementary material for: Viral Metagenomic Content Reflects Seawater Ecological Quality in the Coastal Zone
Source: Viruses. 2020 Jul 26;12(8):806. doi: 10.3390/v12080806 (PMC7472104; doi:10.3390/v12080806)
Supplement: Supplementary file 1 [file viruses-12-00806-s001.pdf]

viruses

MDPI

Supplementary Information

Materials and Methods

Determination of physicochemical and biological parameters

To determine the concentration of chlorophyll, samples (1-2 L) were collected and filtered through 0.47 µm pore size glass microfiber membranes. The membranes were stored at -20 ° C until analysis using a TURNER 00-AU-10 fluorometer (Holm-Hansen *et al.*, 1965). The preservation of the samples by immediate freezing and their frozen storage does not affect the original nutrients concentrations, even those of silicate as 'the silicate polymerisation problem' is not important at the low concentrations (MacDonald et al., 1986; Dore et al., 1996). Nitrate-nitrite and silicate concentrations were measured on a Seal III autoanalyzer according to standard methods (Strickland and Parsons 1977, Mullin and Riley, 1955, respectively). The detection limits for the nitrate and nitrite, nitrite and silicate methods are 0.05 µmol L^{-1}, 0.01 and 0.09 µmol L^{-1}, respectively. TN and TP analysis was performed after a wet-oxidation with persulfate in low alkaline conditions and measured using a SEAL III autoanalyzer (Pujo-Pay and Raimbault, 1994; Raimbault et al., 1999). Values were corrected for the reagent blank. Phosphate and ammonium concentrations were measured with a 25Lambda Perkin Elmer spectrophotometer using standard methods (Murphy and Riley, 1962, Koroleff, 1970, respectively). The detection limits for ammonium and phosphate analysis are 0.034 µmol L^{-1} and 0.003 µmol L^{-1}, respectively. For the determination of particulate organic carbon (POC) concentration, about 2-4 L of seawater - depending on the suspended matter concentration - were filtered through pre-combusted (450º C; 6h), pre-weighed 47 mm diameter, 0.7 µm particle retention GF/F filters, and were analyzed in the laboratory with a Thermo Scientific FLASH 2000 CHNS elemental analyzer according to the methodology suggested by Cutter and Radford-Knoery (1991). Inorganic carbon was removed from the POC filters with repeated *in situ* treatments with HCl 2M. In the laboratory, GF/F filters were lyophilized until dry. The precision of the method is within 5%.

The abundance of virus-like particles (VLP) was determined based on Brussaard (2004) and that of heterotrophic and autotrophic bacteria (HB and AB, respectively) based on Marie *et al.* (1997) from fixed samples with 0.2-µm filtered glutaraldehyde. VLP and HB were diluted in Tris-EDTA buffer solution (pH=8, Sigma-Aldrich) to maintain particles' enumeration at a rate of <1000 events sec^{-1}, stained with SYBR Green I (Molecular Probes) at a 5×10^{-5} and $4x10^{-4}$ final dilution of the stock solution, respectively and then incubated for 10 min at 80 °C and for 10 min in the dark, respectively. Yellow-green latex beads of 1 µm nominal size (Polysciences) were used as an internal standard of fluorescence. A FACSCalibur™ instrument (Becton Dickinson) was used at conventional air pressure, with an air-cooled laser at 488 nm and standard filter setup. The flow rate of the instrument was daily determined and used for abundance conversion, by accurately weighing a trial TRIS-EDTA buffer solution sample before and after running for 5 min at all speed performances. Data were processed with the Cell Quest Pro software (Becton Dickinson).

DNA extraction, amplification, sequencing and sequence processing

Viral dsDNA metagenome preparation started by firstly resuspending the 1 µm filters in ascorbic acid buffer (John *et al.*, 2011). Briefly, a solution of ascorbate-EDTA buffer was prepared daily (0.25 M ascorbic acid, 0.2 M Mg_2EDTA, pH 6–7 adjusted with Tris HCl and NaOH), kept in the dark and added in the viral flocculate, followed by shaking by hand and rotation overnight at 4 °C. After re-suspension, viral particles in liquid were retained from the filter by low-speed centrifuge and then concentrated via ultra-centrifugation at 141.000 g.

Viral DNA extraction was performed with a CTAB protocol (Winnepenninckx et al., 1993); the viral particles were incubated at 60 °C for 2 hours at 2 turns min^{-1} with 10 mL CTAB buffer [2% CTAB (hexadecyltrimethylammonium bromide); 100 mM TrisHCl (pH=8); 20 mM EDTA; 1.4 M NaCl; 0.2% β-mercaptoethanol; 0.1 mg mL^{-1} proteinase K; 10 mM DTT (dithiothreitol)]. DNA was purified using

equal volume of chloroform:isoamylalcohol solution (24:1), followed by centrifuge at 75,000 rpm for 10 min at 4°C. The aqueous phase was treated with RNase and the chloroform:isoamylalcohol step was repeated. DNA was then precipitated with a 2/3 volume of isopropanol overnight, followed by centrifuge at 7500 rpm for 15 min at 4°C to pellet DNA. Pellet was washed with 76% *v/v* ethanol and 10 mM ammonium acetate solution. The extracted DNA was dissolved in ultrapure water and stored at -20°C until PCR amplification and sequencing. For viral DNA quantification the Qubit® high sensitivity assay kit was used in a 3.0 QubitTM fluorometer (Thermo Fisher). Viral DNA shearing was done at 300 bp using the standard protocol for Covaris™ focused ultra-sonicator system. An indexed library for Illumina sequencing was prepared using the NEBNext Ultra DNA Library Prep Kit for Illumina (New England BioLabs®), following manual instructions. Size selection was done using AMPure® XP beads (Beckman Coultier) and PCR cycles were 6 following manufacturer conditions, with regards to the amount of DNA input. Metagenomic libraries were sequenced in the Illumina Hiseq 4000 platform available at KAUST Bioscience Core Lab using paired-end sequencing.

The frozen 0.2-μm filters were grinded with a mortar and pestle in a continuous flow of liquid nitrogen. Grinded filters were treated with the same CTAB protocol described above for DNA extraction. Bacterial DNA quantification and quality control was also done with the same methods. Polymerase Chain Reactions (PCR) were carried out with 25 μL final volume for the 16S rRNA gene, using the locus-specific primers (341f: 5-CCTACGGGNGGCWGCAG-3 and 806RB: 5-GGACTACNVGGGTWTCTAAT -3, Klindworth *et al.*, 2013 and modifications of Apprill *et al.*, 2015) and a universal 5′ tail specified by Illumina (2.5 μl DNA template, 2.5 μL PCR buffer with dNTPs mixture; 10x AccuPrime™ PCR buffer II, 1 μL from each primer; 10 μM and 0.2 μL AccuPrime™ Taq high fidelity DNA polymerase; 1 unit. DNA template concentration was 50-300 ng μL^{-1}. The PCR protocol used was: 98°C for 3 minutes; 28 cycles at 98°C for 30 seconds; 55°C for 30 seconds; 72°C for 30 seconds; 72°C for 5 minutes. The second PCR was done with primers that included the indexes and the Illumina adaptors with 50 μL final volume (5 μl clean DNA template, 10 μL PCR reaction buffer; 5x Q5, New England BioLabs®, 1 μL dNTPs mixture; 10mM, 5 μL from each primer; 10 μM and 0.5 μL Q5® high fidelity DNA polymerase; 0.02 unit μL^{-1}. The PCR protocol used was: 98°C for 3 minutes; 8 cycles at 98°C for 30 seconds; 55°C for 30 seconds; 72°C for 30 seconds; 72°C for 5 minutes. Amplifications were carried out using BIORAD. First-PCR product was cleaned up using the illustra™ ExoProStar™ PCR and Sequence reaction clean-up kit, following manufacturer instructions. The SequalPrep™ Normalization plate kit was used to purify and normalize second-PCR products, following manufacturer instructions. Pooled PCR products were run in an Agilent 2100 Bioanalyzer (Agilent Technologies). PCR products presence and length were ascertained by gel electrophoresis in 1% *w/v* agarose gel.

Viral metagenome reads in FASTQ format were imported to CLC Genomics Workbench v.7 (CLC Bio) and trimmed using a minimum phred score of 20, a minimum length of 50 bp, allowing no ambiguous nucleotides and trimming off Illumina sequencing adaptors if found. The trimmed metagenome reads were assembled using CLC's *de novo* assembly algorithm, using a k-mer of 63 and a minimum scaffold length of 500 bp. The assembled contigs were then analyzed using the iVirus pipeline (Bolduc *et al.*, 2017a) through the Cyverse platform (Goff *et al.*, 2011). Briefly, viral contigs were identified using the VirSorter software, which classifies viral and prophages sequences with three levels of confident predictions. We only considered the first two levels for the rest of the analysis. The vContact software was then used to perform guilt-by-contig-association automatic classification of viral contigs. Viral contigs were also taxonomically assigned with VirFinder (Ren *et al.*, 2017). Viral sequences with a VirFinder score ≥ 0.7 and $p < 0.05$ and VirSorter categories 1 and 2 were used for further analysis. The taxonomy of the viral sequences obtained by the VirFinder software was determined by blasting them against the Global Ocean Viromes 2.0 database (Gregory *et al*, 2019) while the vContact software self-assigned the taxonomy of the viral sequences, Bolduc *et al.*, 2017a).

The list of auxiliary metabolic genes (AMGs) was created by references in Hurwitz and U'Ren (2016), Thompson *et al.* (2011), Puxty *et al.* (2015), Sharon et al. (2011) and Hurwitz *et al.* (2013; 2015). Normalization of gene copy numbers was done using the results of the viral flow cytometry. Phage attachment sites (*attP*) that are exact matches to bacterial tRNA gene (*attB*) (Mizuno *et al.*, 2013) were

obtained by blasting the viral contigs against the tRNADB-CE database with minimum of 100% identity (Abe *et al.*, 2014) as in Bellas *et al.* (2015).

The raw 16S rRNA sequences were quality-checked and analysed using both UPARSE v8 and QIIME v1.9. Paired-end reads were formed with the fastq-join algorithm (https://code.google.com/p/ea-utils/wiki/FastqJoin), by assembling the raw forward and reverse reads of each sample with a minimum overlap of 50 nucleotides and a maximum of one mismatch within the overlapping region. The quality of the paired reads was then checked in QIIME, the forward and reverse primers were removed from the sequence ends of the high-quality reads and the individual sample files were merged. The single file that contained all sample reads was then imported in UPARSE where operational taxonomic units (OTUs) of 97% sequence similarity were picked and chimeric sequences were further discarded by de-novo and reference-based detection. For reference-based detection, the "Gold" database (http://microbiomeutil.sourceforge.net/) was used. The representative sequences of the OTUs were then assigned taxonomy in QIIME with UClust and searching against the newest Greengenes database. Rarefaction curves were drawn indicating that the diversity in all samples was adequately covered. Finally, the OTU counts for each sample and the taxonomic assignments were combined into an OTU table. OTUs that were taxonomically affiliated to Archaea and OTUs without a taxonomic assignment were further removed from subsequent analyses. The resulting OTU table was used as an input for alpha- and beta-diversity analyses.

References

Abe, T., Inokuchi, H., Yamada, Y., Muto, A., Iwasaki, Y., and Ikemura, T. (2014) tRNADB-CE: tRNA gene database well-timed in the era of big sequence data. *Front. Genet.* **5**: 1–7.

Apprill, A., McNally, S., Parsons, R., and Weber, L. (2015) Minor revision to V4 region SSU rRNA 806R gene primer greatly increase detection of SAR11 bacterioplankton. *Aquat. Microb. Ecol.* 7**5**: 129-137.

Bellas, C.M., Anesio, A.M., and Barker, G. (2015) Analysis of virus genomes from glacial environments reveals novel virus groups with unusual host interactions. *Front. Microbiol.* **6**: 1–14.

Bolduc, B., Jang, H. Bin, Doulcier, G., You, Z., Roux, S., and Sullivan, M.B. (2017a) vConTACT : an iVirus tool to classify double-stranded DNA viruses that infect Archaea and Bacteria. *PeerJ* **5**: e:3243.

Bolduc, B., Youens-Clark, K., Roux, S., Hurwitz, B.L., and Sullivan, M.B. (2017) iVirus: Facilitating new insights in viral ecology with software and community data sets imbedded in a cyberinfrastructure. *ISME J.* **11**: 7–14.

Brussaard, C.P.D. (2004) Optimization of Procedures for Counting Viruses by Flow Cytometry. *Appl. Environ. Microbiol.* **70**: 1506–1513.

Cutter, G.A., Radford-Knoery, J., 1991. Determination of carbon, nitrogen, sulfur and inorganic sulfur species in marine particles. *In*: Hurd, D.C., Spencer, D.W. (eds) Marine particles: analysis and characterization. American Geophysical Union, *Geophysical Monograph Series*, 63: 57–63.

Dore, J.E., Houlihan, T., Hebel, D.V., Tien, G., Tupas, L., Karl, D.M., 1996. Freezing as a method of sample preservation for the analysis of dissolved inorganic nutrients in seawater. *Marine Chemistry*, 53, 173-185.

Goff, S.A., Vaughn, M., McKay, S., Lyons, E., Stapleton, A.E., Gessler, D., et al. (2011) The iPlant Collaborative: Cyberinfrastructure for Plant Biology. *Front. Plant Sci.* **2**: 1–16.

Holm-Hansen, O., Lorenzen, C., Holmes, R., and Strickland, J. (1965) Fluorometric determination of chlorophyll. *CIES J Mar Sci* **30**: 3–15.

Hurwitz, B.L. and U'Ren, J.M. (2016) Viral metabolic reprogramming in marine ecosystems. *Curr. Opin. Microbiol.* **31**: 161–168.

Hurwitz, B.L., Brum, J.R. and Sullivan, M. B. (2015) Depth-stratified functional and taxonomic niche specialization in the "core" and "flexible" Pacific Ocean Virome. *The ISME J.* **9:** 472-484.

John, S.G., Mendez, C.B., Deng, L., Poulos, B., Kauffman, A.K.M., Kern, S., et al. (2011) A simple and efficient method for concentration of ocean viruses by chemical flocculation. *Environ. Microbiol. Rep.* **3**: 195–202.

Klindworth, A., Pruesse, E., Schweeer, T., Peplies, J., Quast, C., Horn, M., and Glöckner. O. (2013) Evaluation of general 16S ribosomal RNA gene PCR primers for classical and next-generation sequencing-based diversity studies. *Nucleic Acids Res.* **41 (1):** e1.

Koroleff, F. (1970). Revised version of "Direct determination of ammonia in natural waters as indophenol blue. Int. Con. Explor. Sea C. M. 1969/C:9 ICES information on techniques and methods for sea water analysis. *Interlab. Rep.*, No 3: 19-22.

MacDonald, R.W., MacLaughlin, F.A., Wong, C.S.,1986. The storage of reactive silicate samples by freezing. *Limnol. Oceanogr*., 31, 1139-1142.

Marie, D., Partensky, F., Jacquet, S., and Vaulot, D. (1997) Enumeration and cell cycle analysis of natural populations of marine picoplankton by flow cytometry using the nucleic acid stain SYBR Green I. *Appl. Environ. Microbiol.* **63**: 186–193.

Mizuno, C.M., Rodriguez-valera, F., Kimes, N.E., and Ghai, R. (2013) Expanding the Marine Virosphere Using Metagenomics. *PLOS Genet.* **9**: e1003987.

Mullin, J. B., Riley, J. P., 1955. The colorimetric determination of silicate with special reference to sea and natural waters. *Analytica Chimica Acta*, 12: 162-176.

Murphy, J., Riley, J. P., 1962. A modified solution method for determination of phosphate in natural waters. *Analytica Chimica Acta*, 27: 31-36.

Pavlidou, A., Simboura, N., Rousselaki, E., Tsapakis, M., Pagou, K., Drakopoulou, P., et al. (2015) Methods of eutrophication assessment in the context of the water framework directive: Examples from the Eastern Mediterranean coastal areas. *Cont. Shelf Res.* **108**: 156–168.

Pujo-Pay, M. & Raimbault, P. 1994. Improvement of the wet-oxidation procedure for simultaneous determination of particulate organic nitrogen and phosphorus collected on filters. *Mar. Ecol. Prog. Ser*. 105: 203-207.

Puxty, R.J., Millard, A.D., Evans, D.J., and Scanlan, D.J. (2015) Shedding new light on viral photosynthesis. *Photosynth. Res.* **126**: 71–97.

Ren, J., Ahlgren, N.A., Young Lu Y., Fuhrman, J.A., Sun, F. (2017) VirFinder: a novel k-mer based tool for identidying viral sequences from assembled metagenomics data. *Microbiome* **5:** 69. https://doi.org/10.1186/s40168-017-0283-5

Simboura, N., Pavlidou, A., Bald, J., Tsapakis, M., Pagou, K., Zeri, C., et al. (2016) Response of ecological indices to nutrient and chemical contaminant stress factors in Eastern Mediterranean coastal waters. *Ecol. Indic.* **70**: 89–105.

Sharon, I., Battchikova, N., Aro, E.-M., Giglione, C., Meinnel, T., Glaser, G., et al. (2011) Comparative metagenomics of microbial traits within oceanic viral communities. *The ISME J*. **5:** 1178-1190.

Strickland, J. D. H., Parsons, T. R.,1977. A practical handbook of sea water analysis. Fisheries Research Board of Canada, 167, 310p.

Winnepenninckx, B.T., Backeljau, T., and Wachter, R. De (1993) Extraction of high molecular weight DNA from molluscs. *Trends Genet.* **9**: 407.

Supplementary Tables

Table S1. Sample ID, collection date, water district, longitude and latitude, water body name, maximum depth of the water column and the NCBI BioSample accession numbers for 16S data and viral metagenomes submission.

Sample ID	Collection Date	Water District	Longitude	Latitude	Water Body	Max. depth	BioSample accession ID (16S data)	BioSample accession ID (viral metagenomes)
Arachthos	15/3/2014	Epirus	210,942	39,039,199	North Amvrakikos Gulf	14	SAMN15350032	SAMN15349947
Heraklion	12/4/2014	Crete	25,104,799	353,717	Gulf of Heraklion	79	SAMN15350035	SAMN15349950
Igoumenitsa	8/3/2015	Epirus	20,228,099	39,499,999	Cove of Igoumenitsa (ormos)	18	SAMN15350038	SAMN15349953
Kalamas	18/3/2014	Epirus	20,143,899	39,577,499	Eastern Kerkyra Sea	11	SAMN15350033	SAMN15349948
Kefalonia	9/3/2015	Northern Peloponnese	204,517	38,241,699	Gulf of Argostoli	19	SAMN15350030	SAMN15349945
Louros	19/3/2014	Epirus	20,804,631	39,030,245	North Amvrakikos Gulf	15	SAMN15350034	SAMN15349949
South Amvrakikos	15/3/2014	Epirus	21,094,499	38,921,501	South Amvrakikos Gulf	45	SAMN15350031	SAMN15349946
Saronikos Elefsina	24/3/2015	Attiki	235,575	380,181	Elefsina bay	14	SAMN15350041	SAMN15349955
Saronikos Psittalia	23/3/2015	Attiki	235,958	379,283	Central inner Saronikos Gulf	56	SAMN15350040	SAMN15349954
South Patraikos	6/3/2015	Northern Peloponnese	215,711	381,814	South Patraikos Gulf	62	SAMN15350036	SAMN15349951
West Patraikos	7/3/2015	Northern Peloponnese	21,510,000	38,250,000	West Patraikos Gulf	76	SAMN15350037	SAMN15349952
Messolonghi	3/7/2015	Western Central Greece	21.410.999	38.302.101	Messolonghi Sea	42	SAMN15350029	SAMN15349944
Messiniakos	3/12/2014	Western Peloponnese	22.090.999	36.997.398	Gulf of Kalamata	167	SAMN15350028	SAMN15349943
Saronikos Epidavros	3/5/2015	Attiki	23.255.599	37.647.201	West Saronikos Gulf	404	SAMN15350027	SAMN15349942

Table S2. Sample ID and the four measurements used for the calculation of the annual TRIX, i.e. the monthly percentage deviation from oxygen saturation as well as the monthly surface concentrations of phosphate (μg L^{-1}), dissolved inorganic nitrogen (μg L^{-1} as the sum of ammonium, nitrate and nitrite) and chlorophyll *α* (μg L^{-1}). The last three columns present the resulting surface-waters annual TRIX: values, categories and group.

Station ID	Month	DO Dev%	PO4	DIN	Chl *α*	Surface-waters annual TRIX category	Surface-waters annual TRIX value	TRIX group
Arachthos	*March 2014*	11.74	4.19	9.87	0.12	Moderate	3.73	MoPoB
	September 2014	3.61	0.48	5.44	0.57			
	December 2014	8.73	7.23	117.26	1.84			
	March 2015	15.06	2.25	12.18	2.70			
	July 2015	5.19	1.31	4.62	0.62			
Heraklion	*March 2014*	0.14	2.85	9.08	0.24	Good	2.22	Good
	September 2014	2.61	1.26	15.94	0.06			
	December 2014	0.32	5.59	8.80	0.11			
	March 2015	1.66	3.36	55.27	0.35			
	July 2015	4.34	0.48	4.62	0.05			
Igoumenitsa	March 2014	4.99	1.63	47.34	0.23	Moderate	3.83	MoPoB
	September 2014	8.95	3.87	73.50	0.92			
	December 2014	5.15	8.87	152.45	0.52			
	March 2015	0.27	1.71	60.43	1.10			
	July 2015	11.13	0.48	4.62	1.74			
Kalamas	*March 2014*	5.83	5.73	22.58	0.22	Moderate	3.25	MoPoB
	September 2014	10.61	1.78	37.65	0.21			
	December 2014	7.28	22.01	34.48	0.31			
	March 2015	5.89	7.75	14.00	0.34			
	July 2015	4.02	4.34	20.86	0.28			
Kefalonia	March 2014	0.20	3.17	79.01	0.62	Good	2.62	Good
	September 2014	2.01	2.82	12.92	0.71			
	December 2014	0.43	3.39	80.00	0.92			
	March 2015	1.75	2.25	14.53	0.94			
	July 2015	4.80	1.81	4.62	0.44			
Louros	*March 2014*	12.80	7.26	49.47	0.06	Poor	4.46	MoPoB
	September 2014	3.42	1.26	12.11	1.48			
	December 2014	0.76	12.16	150.19	2.30			
	March 2015	16.58	3.91	67.07	11.34			
	July 2015	0.50	2.82	6.63	1.59			
South Amvrakikos	*March 2014*	10.82	3.17	4.10	0.17	Moderate	3.15	MoPoB
	September 2014	3.14	1.78	18.43	0.29			
	December 2014	1.12	5.04	31.66	1.27			
	March 2015	1.32	3.90	6.81	2.75			
	July 2015	4.86	1.81	7.68	0.73			
Saronikos Elefsina	March 2014	11.78	1.63	26.98	0.57	Poor	4.22	MoPoB
	September 2014	1.07	1.78	57.26	1.46			
	December 2014	11.08	15.44	179.84	0.66			
	March 2015	2.00	5.56	305.50	0.64			
	July 2015	11.31	3.33	16.73	0.49			
Saronikos Psittalia	March 2014	6.82	5.73	31.28	0.16	Moderate	3.52	MoPoB
	September 2014	5.33	2.82	9.97	0.35			
	December 2014	2.72	11.06	95.69	0.51			
	March 2015	2.38	11.61	61.06	0.64			
	July 2015	9.31	3.83	5.68	0.07			
South Patraikos	March 2014	2.19	0.48	31.66	0.16	Good	2.12	Good
	September 2014	2.41	1.25	4.17	0.09			
	December 2014	0.75	2.30	14.20	0.19			
	March 2015	4.87	13.25	89.19	0.43			
	July 2015	0.89	0.48	12.57	0.11			
West Patraikos	March 2014	1.79	1.12	32.23	0.23	Good	2.58	Good
	September 2014	2.84	1.78	5.62	0.07			
	December 2014	1.93	2.30	31.99	0.25			

	March 2015	3.75	8.30	68.63	0.33			
	July 2015	1.02	0.48	9.40	0.18			
Messolonghi	March 2014	4.33	2.15	10.45	0.16	Good	2.21	Good
	September 2014	1.87	1.78	12.14	0.10			
	December 2014	1.28	2.30	15.68	0.20			
	March 2015	1.50	2.25	59.81	0.33			
	July 2015	1.22	0.48	7.49	0.12			
Messiniakos	*March 2014*	0.35	3.69	27.15	0.21	Good	2.19	Good
	September 2014	2.45	1.78	19.58	0.13			
	December 2014	0.81	2.30	15.22	0.17			
	March 2015	-	-	-	-			
	July 2015	5.78	0.48	4.62	0.15			
Saronikos Epidavros	March 2014	3.07	1.63	14.65	0.01	Good	2.14	Good
	September 2014	1.58	2.30	3.96	0.18			
	December 2014	2.19	2.30	15.39	0.17			
	March 2015	6.73	2.80	4.01	0.40			
	July 2015	5.90	1.31	7.78	0.03			

Table S3. The raw numbers of viral metagenome reads, the quality of reads, the number of total contigs, the contigs-length (total, minimum and maximum), and the N50 contig length.

Station ID	Raw reads	Quality reads	Number of contigs	Total contigs length	Minimum length	Maximum Length	Contigs N50
Arachthos	33507098	24274378	18243	48245552	985	88035	3140
Heraklion	70231892	51089443	34036	106002782	962	1107943	4030
Igoumenitsa	43150468	32331404	32079	77298434	981	144765	3014
Kalamas	36024010	26008487	27467	65377333	880	96291	2599
Kefalonia	29518322	19126722	13640	29149669	975	61662	2223
Louros	33955578	26666081	18447	57084554	911	366376	4226
South Amvrakikos	48302208	31538764	20077	47112620	965	66530	2536
Saronikos Elefsina	37582022	27345555	19108	44401617	871	136287	2413
Saronikos Psittalia	23690854	12401698	8331	15381461	1000	93622	1766
South Patraikos	31789304	17571115	10264	25606220	982	207035	2662
West Patraikos	40463604	28090883	11560	31981640	960	492488	3331
Messolonghi	22645972	10468773	3338	7044119	1001	62445	2203
Messiniakos	26638740	16704702	8605	25603976	998	367646	3826
Saronikos Epidavros	17542228	7208702	3790	6660343	1001	35552	1686

Table S4. Abundance matrix of the taxonomically-annotated viral lytic contigs at the family level.

Station ID	*Podoviridae*	*Myoviridae*	*Siphoviridae*	*Phycodnaviridae*	*Inoviridae*
Arachthos	357.0	315.0	114.0	19.0	0.0
Heraklion	233.0	335.0	68.0	2.0	0.0
Igoumenitsa	547.0	419.0	163.0	66.0	1.0
Kalamas	413.0	433.0	150.0	13.0	0.0
Kefalonia	329.0	267.0	97.0	6.0	1.0
Louros	307.0	295.0	129.0	5.0	0.0
South Amvrakikos	184.0	181.0	53.0	7.0	0.0
Saronikos Elefsina	484.0	622.0	260.0	21.0	0.0
Saronikos Psittalia	52.0	25.0	21.0	0.0	0.0
South Patraikos	52.0	95.0	21.0	16.0	1.0
West Patraikos	74.0	130.0	23.0	34.0	0.0
Messolonghi	34.0	51.0	19.0	12.0	0.0
Messiniakos	242.0	210.0	68.0	1.0	0.0
Saronikos Epidavros	73.0	41.0	11.0	0.0	0.0

Table S5. Abundance matrix of the 67 tested AMGs (normalized abundance with the flow cytometry results of virus-like particles) along with their potential functional role. Photosynth: AMGs involved in host photosynthesis, Energy: AMGs involved in host energy production and dNTP synthesis. Other: AMGs involved in various host metabolic functions. The normalization factors for the AMG copy numbers for Arachthos, Heraklion, Igoumenitsa, Kalamas, Kefalonia, Louros, South Amvrakikos, Saronikos Elefsina, Saronikos Psittalia, South Patraikos, West Patraikos, Messolonghi and Saronikos Epidavros were: 7.9, 1.2, 2.8, 1.9, 2.1, 10.1, 7.8, 8.1, 2.4, 1.2, 1.0 (minimum), 1.9 and 1.8, respectively.

**AMG**	**Functional potential**	**Arachthos**	**Heraklion**	**Igoumenitsa**	**Kalamas**	**Kefalonia**	**Louros**	**South Amvrakikos**	**Saronikos Elefsina**	**Saronikos Psittalia**	**South Patraikos**	**West Patraikos**	**Messolonghi**	**Saronikos Epidavros**
ACC	Other	32997	42423	43690	15181	20467	93009	101995	41997	12183	15253	10964	4728	10737
ATP_syn	Energy	35412	35364	24243	13690	11577	3144	62215	26258	8095	7160	4814	2202	6406
cpet	Photosynth	16	0	0	6	0	448482	16	89	0	0	0	0	2
CytC	Other	89236	93846	106810	40146	50263	140456	228967	95138	26741	35302	24005	9757	25699
aceAB	Energy	689	572	493	300	214	48243	2160	859	135	160	288	127	83
aceEF	Energy	736	1152	354	242	144	1924	819	446	205	114	51	20	49
acn	Energy	6604	7600	4853	2727	2268	30	11729	5292	1540	1540	1079	424	1196
cp12	Energy	475	119	108	138	316	0	242	721	24	117	113	179	65
dsrC	Other	538	882	408	209	208	4242	499	348	147	102	64	19	96
eda	Energy	6216	9446	3572	1939	1363	41522	6473	3080	1437	1257	524	246	766
edd	Energy	5060	9737	4110	2062	1519	22056	7252	3096	1405	1239	785	395	958
eno	Energy	12512	12118	9774	4740	4957	28182	25852	11435	2970	3234	2847	1298	2594
fadL	Other	158	42	48	107	6	68283	86	105	22	16	16	2	5
fadb	Other	14950	22041	22885	7293	9996	13209	52842	21744	6955	7835	5923	2143	5129
fba	Energy	5401	2509	3243	1760	1890	29200	9077	3258	1164	981	704	305	1038
fum	Other	8837	6031	6696	2933	2884	19698	17297	8453	1957	2207	1999	691	1514
gap	Energy	14705	19389	13040	5779	6576	45986	28300	12894	4505	4255	2677	1229	3409
glgA	Energy	2043	5177	1908	1518	585	16020	5357	4312	728	507	690	125	328
gltA	Energy	26551	28313	22049	10215	10346	56817	59845	25586	6152	7009	6316	2506	5342
gnd	Energy	1299	991	720	454	1488	2761	1279	932	294	593	774	1389	632
gpm	Energy	5060	4388	3566	1894	1827	20625	10489	3266	1094	1173	972	467	875
hli	Photosynth	1077	734	1159	762	2111	8282	2987	2115	612	561	599	962	826
ho1	Photosynth	356	9	23	92	2	111	109	1037	5	9	20	4	4
icd	Energy	17777	19242	17684	6650	9219	40202	35498	17109	5496	5081	3542	1659	4159
manA	Energy	626	438	167	150	119	13330	819	219	36	27	33	9	34
mcm	Other	10057	18904	18327	6722	8369	30731	40848	21136	3828	6118	6497	1999	4261
ndhD	Other	2407	2306	2767	1210	1193	8272	6434	3566	617	885	1190	240	531

ndhL	Other	380	550	533	197	229	907	1466	478	222	222	129	28	89
ndhP	Other	24	5	6	4	2	10	0	8	2	0	1	0	9
pcc	Other	8386	9019	10985	3639	5012	8313	24994	10495	2266	3820	3702	1156	2297
pck	Energy	5599	4605	1548	1460	759	22943	8828	2172	417	506	263	173	322
pcyA	Photosy nth	32	0	9	19	0	0	31	251	0	0	0	0	2
pebS	Photosy nth	24	46	68	47	11	594	39	0	10	3	6	9	29
petE	Photosy nth	436	17	23	123	15	151	156	1045	48	7	7	13	80
petF	Photosy nth	523	110	130	138	76	514	172	1386	55	21	15	32	105
pfk	Energy	4957	2181	1959	1647	945	11083	7190	3501	557	500	979	169	409
pgi	Energy	7808	11830	6954	3610	2861	33734	16876	6824	2336	2251	1759	549	1505
pgk	Energy	10754	10978	8278	3925	4264	25270	23356	9904	2685	2893	2433	1132	2243
pgl	Energy	1251	1630	788	407	292	1219	1427	713	224	205	128	41	120
ppc	Energy	9795	11162	7359	4403	3757	45270	20728	8639	3532	2265	1339	866	2297
pps	Energy	11672	17385	13545	6566	6336	51215	29369	14126	3956	4385	2055	1070	3663
psaA	Photosy nth	214	269	329	99	237	141	359	122	94	52	66	7	43
psaB	Photosy nth	190	438	539	189	324	3123	671	494	140	124	56	60	103
psaC	Photosy nth	24	9	167	6	110	5864	257	81	27	52	11	9	47
psaD	Photosy nth	214	322	156	160	51	2207	281	146	58	37	27	19	31
psaE	Photosy nth	0	1	3	2	2	0	0	0	0	0	0	0	0
psaF	Photosy nth	0	6	3	0	6	0	8	0	0	0	0	0	2
psaJ	Photosy nth	0	0	9	0	4	0	0	8	0	0	0	0	4
psaK	Photosy nth	0	0	0	4	0	0	16	0	0	0	0	0	0
psbA	Photosy nth	12258	3858	6100	4418	11798	39799	18505	7942	3951	3622	3801	6527	5458
psbD	Photosy nth	1766	967	1029	961	2725	7073	2113	1742	456	1067	1275	2969	810
ptoX	Photosy nth	40	12	3	4	8	60	16	49	27	1	0	7	125
pyk	Energy	7642	6855	5590	2604	2641	21613	16673	6816	1615	1940	1802	726	1281
queuos ine	Other	29085	38403	30695	12836	15672	12258 2	74537	39453	10322	10670	7896	4179	8364

rdsr	Other	277	57	224	37	93	181	187	105	67	74	22	34	40
rpe	Energy	820163	945872	909618	33697<br>9	393414	26748<br>04	2214654	1021470	238771	295879	271005	102349	201405
rpi	Energy	3627	3560	2852	1436	1418	14388	7291	2877	981	937	585	216	716
sdh	Other	475	1375	448	390	165	4192	1427	413	123	164	125	54	150
sucA	Energy	20391	19434	16742	7882	8297	50973	44224	18713	4780	5408	4687	2115	4326
sucB	Energy	11728	12255	9003	4247	4442	25572	24011	9231	2485	2932	2267	845	2219
sucC	Energy	10453	11023	9896	4050	5050	25703	23543	10358	2743	3258	2626	1245	2663
succD	Energy	7840	7488	6738	2949	3410	16726	16759	7399	1916	2186	1770	778	1598
tal	Energy	7071	7077	5298	2460	2592	32504	15098	6257	1815	1735	1456	683	1370
talC	Energy	2075	714	1338	885	1926	7124	1373	3582	458	532	728	1331	723
tkt	Energy	13929	15621	12164	5600	5421	55689	35381	12546	4076	4476	3549	1499	3007
tpi	Energy	4870	3926	2914	1653	1339	14721	8446	3355	947	829	758	218	750
zwf	Energy	1980	1115	853	641	1170	1491	1599	3242	345	351	298	605	600

Table S6. Results of the one-way analysis of variance (ANOVA) of the normalized abundance of AMGs between groups MoPoB and Good (only the significantly different AMGs are presented, Tukey HSD, p<0.1).

AMG	Sum of Squares	df	Mean Square	F	Sig.
ACC	3163739399	11	3163739399	4.112	.068
CytC	13281244455	11	13281244455	4.609	.055
eno	269305885	11	269305885	4.549	.056
fum	151852393	11	151852393	5.734	.036
gltA	1244961651	11	1244961651	4.418	.059
icd	538398347	11	538398347	4.691	.053
mcm	399191203	11	399191203	3.499	.088
ndhD	21057820	11	21057820	4.712	.053
ndhL	491230	11	491230	3.894	.074
petF	411849	11	411849	3.434	.091
pfk	40705074	11	40705074	5.302	.042
pgk	208540603	11	208540603	4.244	.064
pyk	132132070	11	132132070	4.082	.068
queuosine	3194931723	11	3194931723	3.383	.093
rdsr	32906	11	32906	7.381	.020
rpe	2096009096062	11	2096009096062	4.135	.067
sucA	827991680	11	827991680	4.440	.059
sucB	215401648	11	215401648	4.081	.068
sucC	211003802	11	211003802	4.298	.062
succD	106687212	11	106687212	4.810	.051
tpi	50891456	11	50891456	3.746	.079

Table S7. Reports of distance-based Redundancy Analysis (dbRDA) of the distance-based linear model of the physicochemical variables fitted to (A) the viral community composition (family level), (B) the bacterial community composition (family level) and (C) the bacterial community composition (phylum level).

A – Viral family level

SEQUENTIAL TESTS

Variable	Adj R^2	SS(trace)	Pseudo-F	P	Prop.	Cumul.	res.df
+NO3	0.22557	231.7	4.7866	0.031	0.28514	0.28514	12
+NH4	0.27728	83.961	1.8586	0.18	0.10333	0.38847	11
+T	0.28548	50.3	1.1262	0.321	6.1902E-2	0.45037	10
+NO2	0.28948	46.911	1.0563	0.365	5.7731E-2	0.5081	9
+TN	0.31755	58.451	1.3702	0.307	7.1932E-2	0.58003	8
+PP	0.46275	106.19	3.162	0.09	0.13068	0.71071	7
+PO4	0.56222	70.89	2.5906	0.136	8.724E-2	0.79795	6
+TP	0.76277	90.042	6.0723	0.025	0.11081	0.90876	5
+Chl *α*	0.79502	22.89	1.7865	0.287	2.8169E-2	0.93693	4
+DO	0.85517	24.094	2.6616	0.178	2.9651E-2	0.96658	3
+SiO4	0.91464	16.486	3.0898	0.176	2.0289E-2	0.98687	2
+C	0.941	6.9833	1.8935	0.207	8.594E-3	0.99546	1

BEST SOLUTION

Adj R^2	R^2	RSS	No.Vars	Selections
0.941	0.99546	3.688	12	1,2,5-13,15

Percentage of variation explained by individual axes

	% explained variation out of fitted model		% explained variation out of total variation	
Axis	Individual	Cumulative	Individual	Cumulative
1	78.88	78.88	78.52	78.52
2	15.52	94.4	15.45	93.97
3	11.98	106.38	11.93	105.9
4	3.49	109.87	3.48	109.38
5	1.36	111.23	1.35	110.73
6	0.55	111.79	0.55	111.28
7	0	111.79	0	111.28
8	-0.06	111.73	-0.06	111.22
9	-0.69	111.04	-0.68	110.54
10	-1.55	109.49	-1.55	108.99
11	-3.44	106.05	-3.43	105.57
12	-6.05	100	-6.02	99.55

B- Bacterial family level

SEQUENTIAL TESTS

Variable	Adj R^2	SS(trace)	Pseudo-F	P	Prop.	Cumul.	res.df
+C	8,6561E-2	1578,6	2,2319	0,047	0,15683	0,15683	12
+Chl *α*	0,15056	1252,5	1,9042	0,093	0,12442	0,28125	11
+NO2	0,17147	819,61	1,2776	0,316	8,1422E-2	0,36267	10
+PP	0,21946	975,96	1,6148	0,171	9,6954E-2	0,45962	9
+NO3	0,26747	901,83	1,5899	0,182	8,959E-2	0,54921	8
+TN	0,313	814	1,5302	0,191	8,0865E-2	0,63008	7
+SiO4	0,32599	592,28	1,1349	0,346	5,8839E-2	0,68892	6

BEST SOLUTION

Adj R^2	R^2	RSS	No.Vars	Selections
0,32599	0,68892	3131,4	7	2;6;8;9;11;13;15

Percentage of variation explained by individual axes

	% explained variation out of fitted model		% explained variation out of total variation	
Axis	Individual	Cumulative	Individual	Cumulative
1	50,33	50,33	34,68	34,68
2	14,96	65,29	10,31	44,98
3	14,3	79,59	9,85	54,83
4	8,1	87,7	5,58	60,42

5	5,7	93,39	3,92	64,34
6	3,75	97,14	2,58	66,92
7	2,86	100	1,97	68,89

C – Bacterial phylum level

SEQUENTIAL TESTS

Variable	Adj R^2	SS(trace)	Pseudo-F	P	Prop.	Cumul.	res.df
+C	0.11466	327.68	2.6836	0.033	0.18276	0.18276	12
+SiO4	0.18937	235.45	2.1059	0.094	0.13132	0.31408	11
+NH4	0.19459	119	1.0713	0.334	6.6373E-2	0.38045	10
+NO2	0.21378	134.9	1.2441	0.292	7.524E-2	0.45569	9
+NO3	0.27989	181.38	1.8263	0.161	0.10116	0.55686	8

BEST SOLUTION

Adj R^2	R^2	RSS	No.Vars	Selections
0.27989	0.55686	794.53	5	2,6,8-10

Percentage of variation explained by individual axes

	% explained variation out of fitted model		% explained variation out of total variation	
Axis	Individual	Cumulative	Individual	Cumulative
1	47.51	47.51	26.46	26.46
2	22.21	69.73	12.37	38.83
3	17.43	87.15	9.7	48.53
4	10.79	97.94	6.01	54.54
5	2.06	100	1.15	55.69

Table S8. *attP* gene copies and host taxonomic assignment of *attP* to bacterial phyla. Phage attachment sites (*attP*) that are exact matches to bacterial tRNA gene (*attB*) were obtained by blasting viral contigs against the tRNADB-CE database with minimum of 100% identity.

**Station ID**	*Bacteroidetes*	*Chlorobi*	*Cyanobacteria*	*Deinococcus-Thermus*	*Firmicutes*	*Spirochaetes*	*Synergistetes*	*Proteobacteria*
Arachthos				1	1		4	
Heraklion		2		1			1	
Igoumenitsa	1	1						4
Kalamas	1	1			1			0
Kefalonia	2		1					6
Louros				2		1	2	
South Amvrakikos							1	
Saronikos Elefsina				1				1
Saronikos Psittalia	1							2
SouthPatraikos							0	
WestPatraikos							2	
Messolonghi							5	
Saronikos Epidavros								8
								2
